# Bayesian Integrated Learning of Longitudinal Dose‐Response Relationships via Decentralized Clinical Trials

**DOI:** 10.1002/sim.70338

**Published:** 2025-12-03

**Authors:** Jingyi Zhang, Tuo Wang, Yongming Qu, Fangrong Yan, Suyu Liu, Ruitao Lin

**Affiliations:** ^1^ Research Center of Biostatistics and Computational Pharmacy China Pharmaceutical University Nanjing China; ^2^ Department of Global Statistical Sciences Eli Lilly and Company Indianapolis Indiana USA; ^3^ Department of Biostatistics The University of Texas MD Anderson Cancer Center Houston Texas USA

**Keywords:** data integration, decentralized clinical trial, dose‐response model, phase II trial, spike‐and‐slab prior

## Abstract

Decentralized clinical trials (DCTs) extend trial activities beyond traditional sites, enhancing access, convenience, efficiency, and result generalizability. They are particularly promising for chronic conditions like diabetes and obesity, which require longer study durations to evaluate drug effects. However, decentralized data collection raises concerns about increased variability and potential biases. This paper presents a novel Bayesian integrated learning procedure to analyze dose‐response relationships using longitudinal data from a phase II DCT that combines centralized and decentralized data collection. We generalize a parametric exponential decay model to handle mixed data sources and apply Bayesian spike‐and‐slab priors to address biases and uncertainties from decentralized measurements. Our model enables data‐adaptive integration of information from both centralized and decentralized sources. Through simulations and sensitivity analyses, we show that the proposed approach achieves favorable performance across various scenarios. Notably, the method matches the efficiency of traditional trials when decentralized data collection introduces no additional variability or error. Even when such issues arise, it remains less biased and more efficient than naïve methods that rely solely on centralized data or simply pool data from both sources.

## 1 Introduction

Decentralized clinical trials (DCTs) refer to clinical trials that include decentralized elements where trial‐related activities occur at locations other than traditional clinical trial sites [1]. This is typically achieved by leveraging digital technologies and innovative methods, such as remote monitoring devices, electronic patient‐reported outcomes, telehealth consultations, and home‐based visits by healthcare professionals. Compared to traditional clinical trials, DCTs offer greater convenience for participants and caregivers and provide access to more diverse patient populations, particularly those in remote or underserved areas, accelerating patient recruitment. DCTs also reduce the burden on clinicians, accelerate patient recruitment, and improve overall trial efficiency [2]. Additionally, the increased connection between patients and clinical investigators enables enriching datasets through more frequent or even continuous data collection in real‐world settings [3].

DCTs can be fully decentralized, with all activities conducted remotely, or follow a hybrid model, where some activities require in‐person visits to traditional trial sites while others occur at alternative locations, such as participants' homes, local clinics, local pharmacies, or mobile clinical units. The hybrid DCT model is particularly well‐suited for trials with longitudinal endpoints and extended follow‐up periods, and evaluating longitudinal response is critical in certain disease areas. Leveraging the advantages of DCTs, participants can complete the majority of visits remotely while requiring in‐person visits for several key assessments, such as the first and the last visits. This approach not only reduces the burden on both clinicians and participants but also improves patient adherence. Evaluating longitudinal response is critical in certain disease areas for assessing the relationship between treatment and the development of disease [4]. For example, diabetes and obesity treatments typically require several weeks or months to assess drug efficacy. Another example is chronic kidney disease, where proteinuria, a clinical endpoint that requires repeated measurement, is used in dose‐finding trials for early‐phase efficacy evaluation [5].

Despite these advantages, DCTs present several challenges. Ensuring participant safety is one of the most significant concerns, particularly when physical examinations and face‐to‐face interactions are limited [3]. Operationally, securing data storage and transmission poses additional risks. Decentralized data collection also introduces statistical concerns about data quality, including increased variability, potential biases or measurement errors, and issues related to the accuracy and validation of collected data [6]. Regulatory requirements may vary across countries or regions, complicating the implementation of DCTs. Challenges such as bridging the digital divide and achieving regulatory acceptance of digital endpoints persist [7]. To the best of our knowledge, statistical methods to address these emerging challenges, particularly increased variability and potential measurement errors in DCTs, are notably lacking.

In this paper, we focus on the hybrid DCT model [8, 9] in which the same patient may have both onsite and offsite measurements. Operationally, the distinction between DCTs and multicenter trials lies in trial conduct: DCTs allow certain patient activities, including data collection, to occur remotely, whereas multicenter trials rely entirely on in‐person site infrastructure across multiple centers. Statistically, the key difference is in the assumption and treatment of heterogeneity. In multicenter trials, heterogeneity arises primarily from regional differences, such as epidemiology, medical practices, or population‐specific drug metabolism. No single site's data are regarded as inherently more reliable, and the estimand is the average treatment effect across centers, typically modeled with center‐specific random effects. By contrast, DCTs introduce a different source of heterogeneity: decentralized measurements may carry additional uncertainty or bias because they lack centralized quality monitoring [1]. Consequently, hybrid DCTs typically regard centralized data as the primary basis for defining the estimand. The methodological challenge, however, is that each patient also provides decentralized measurements, raising the important question of whether incorporating these data can improve the efficiency of estimation.

In practice, analyses of hybrid DCTs often pool onsite and offsite data without distinction, a practice that can obscure source‐specific heterogeneity and compromise validity—an issue also supported by our simulation studies. As a real‐trial example, Figure 3 of a recent phase 1b/2a randomized trial in overweight or obese patients [10] demonstrated a “zig‐zag” pattern in the weight‐loss curve, largely attributable to differences between onsite and at‐home data collection. Similarly, a simulation study [6] showed that failing to account for such mixed data heterogeneity can lead to substantial bias. This paper aims to fill this gap by developing an innovative Bayesian integrated learning method for analyzing hybrid DCTs, integrating both traditional centralized and novel decentralized data measurements. Specifically, we consider a phase II dose‐ranging decentralized trial for a chronic condition, where a clinical efficacy endpoint is collected longitudinally through both centralized and decentralized methods. In such trials, fully evaluating the longitudinal dose‐response relationship and carefully selecting an appropriate dose are pivotal to the success of drug development [11]. Due to the relatively small or moderate sample sizes for phase II dose‐ranging trials, the Emax model is one of the most commonly used models to characterize the dose‐response relationships [12, 13]. Additionally, dose‐response modeling can also be based on pharmacokinetic and pharmacodynamic characteristics to enhance precision [14], with the appropriate model form tailored to the specific drug. To evaluate the dose‐response relationship with repeated measurements, Fu and Manner [15] proposed the parametric integrated two‐components prediction (ITP) model (also referred to as exponential decay model), which was further developed by Duan et al. [16]; Li and Fu [17]; Payne et al. [18]; Qu [19]. The family of ITP models has been applied in several real‐world phase II trials, for example, see Frias et al. [20]. While our paper focuses on the ITP model, we acknowledge the availability of other longitudinal dose‐response models [21, 22, 23, 24, 25] and believe that our proposed approach could also be applied to these models.

In some clinical trials, certain measurements can be collected frequently through a method at home or locally and infrequently on site. For example, body weight can be measured weekly at home via a standardized scale and monthly on site. It is an important question of how to utilize both types of measurements in estimating the dose‐response relationship in phase 2 studies. Research showed the remote weight measurements could be subject to some bias and increased variability [26]. This paper develops a novel statistical model for hybrid DCTs, combining longitudinal data evaluation with dose‐response analysis and providing flexibility to address biases and uncertainties arising from decentralized data collection. Using the parametric ITP model as an example, we extend it to account for the unique characteristics and challenges posed by decentralized measurements. Additionally, we propose a Bayesian integrated learning approach with spike‐and‐slab priors to adaptively adjust for biases and uncertainties associated with decentralized data.

The remainder of the paper is organized as follows: In Section 2, we review the ITP model and introduce the proposed approach for multidose longitudinal trials with outcomes collected in a hybrid data collection model. Section 3 applies the proposed method to reanalyze the motivating trial. In Section 4, we assess the operating characteristics of the proposed model through simulation studies and perform sensitivity analyses to evaluate the robustness of the design. Finally, we conclude with a discussion in Section 6. Additional simulation details and the R code for implementing the proposed method are provided in the Supporting Information.

## 2 Methods

### 2.1 The Integrated Two‐Component Prediction (ITP) Model

Consider a phase II multidose randomized trial with a longitudinal endpoint measured at L preplanned (standardized) time points: $t_{0}=0<t_{1}<\cdots <t_{L}=1$. Following the motivating trial for weight management, the endpoint $Y_{jl}$ represents an observation (i.e., change from baseline $t_{0}$) for subject j at the lth time $t_{lj}$ (j=1,…,J, l=1,2,…,L) and a negative $Y_{jl}$ is expected for an effective treatment. Typically, $t_{lj}$ does not exactly match $t_{l}$ and may fall within the interval $\left[t_{l}-\epsilon ,t_{l}+\epsilon \right]$, where ϵ denotes the visit tolerance, such as three days. If no observation is collected within this interval, $Y_{jl}$ is treated as missing. In a Bayesian framework, ignorable missingness can be effectively handled using Bayesian data augmentation (BDA) (see further details in Section 2.4). Assume that each patient is randomized to one of the prespecified dose arms $d_{j}\in \{0,\ldots ,D\}$, with $d_{j}=0$ indicating the placebo, and d=1,…,D denoting active doses. Let $m_{d}\geq 0$ indicate the corresponding dosage of d, where $m_{0}=0$. The standard ITP model comprises two components: the time‐effect (i.e., $\kappa (l,d_{j};\theta_{1})\in [0,1]$) and the terminal dose effect (i.e., $\lambda (d_{j};\theta_{2})$), such that

(1) $$Y_{jl}=\kappa (l,d_{j};\theta_{1})(\lambda (d_{j};\theta_{2})+s_{j}+e_{jl}),$$

where $s_{j}∼N(0,\sigma_{s}^{2})$ is the between‐subject error and $e_{jl}∼N(0,\sigma_{e}^{2})$ is the within‐subject error, and $s_{j}$ and $e_{j1},\ldots e_{jL}$ are mutually independent.

Based on empirical results from real‐world studies, [19] further modifies model (1) by assuming that the between‐subject variance depends on the mean response, while the within‐subject variance is independent of it, which was found to provide a better fit to real data. The modified ITP model is expressed as

(2) $$\begin{array}{ll} & Y_{jl}=\mu (l,d_{j};\theta )+\left|\frac{\mu (l,d_{j};\theta )}{\mu (L,d_{j};\theta )}\right|s_{j}+e_{jl},\\ & \mu (l,d_{j};\theta )=\kappa (l,d_{j};\theta_{1})\lambda (d_{j};\theta_{2}), \end{array}$$

where μ(l,d;θ) indicates the mean response at dose d at $t_{l}$.

In the ITP models, the time‐effect $\kappa (l,d;\theta_{1})$ quantifies the percentage of the response achieved at $t_{l}$ and is modeled using a parametric approach:

$$\kappa (l,d_{j};\theta_{1})=\frac{1-\exp(-k(d_{j})t_{l})}{1-\exp(-k(d_{j})t_{L})},$$

where k(d) is the rate parameter that determines how fast the response changes over time for dose d, and $\theta_{1}=\{k(1),\cdots \;,k(D)\}$. Generally, a positive k(d) reflects a drug's rapid initial effect, followed by a plateau over time. Therefore, a larger k(d) suggests that patients are more likely to experience a quick response shortly after treatment, with the effect reaching a plateau earlier. The function $\lambda (d;\theta_{2})$ quantifies the relationship between the dose and the maximum response, achieved at the terminal time $t_{L}$. Depending on the efficacy outcome and the characteristics of the investigational drug, $\lambda (d;\theta_{2})$ can take various forms, such as the Emax model or a log‐linear model [18]. Based on recent findings on weight loss with novel treatments, the drug effect appears to reach a plateau at high doses [27]. Therefore, we adopt the three‐parameter Emax model for $\lambda (d;\theta_{2})$, formally,

(3) $$\lambda (d_{j};\theta_{2})=\phi_{0}+\frac{\phi_{1}m_{d_{j}}}{\phi_{2}+m_{d_{j}}},$$

where $\phi_{0}$ and $\phi_{1}$ denote the placebo and maximum dose effects at time $t_{L}$, $\phi_{2}$ is the dose that produces half of the maximum dose effect, and $\theta_{2}=(\phi_{0},\phi_{1},\phi_{2})$. When necessary, the three‐parameter Emax model can be extended to a four‐parameter Emax model by adding a Hill parameter to control the steepness of the dose–response curve. The meta‐analysis by Thomas et al. [28] demonstrated that the Emax model generally provides a good fit to real‐world dose‐response data across a wide range of pharmaceutical trials.

### 2.2 The Proposed DCT‐ITP Model

Let $I_{l}=1$ indicate a decentralized measurement is collected at the l‐th visit, and $I_{l}=0$ indicate a centralized measurement. Without confusion, we use the terms “centralized” and “onsite” interchangeably to refer to data collected through the traditional centralized approach with formal supervision, whereas “decentralized,” “offsite,” and “remote” are used interchangeably to denote data collected through decentralized approaches with less supervision. If $I_{l}=0$ for all visits, the trial reduces to a traditional clinical trial, with all prespecified visits conducted at the clinical center. We generalize the modified ITP model (2) by introducing additional parameters to account for the bias and uncertainty introduced by DCTs and to model the potential differences between centralized and decentralized measurements. Specifically, the DCT‐ITP model is proposed as follows

(4) $$\begin{array}{ll} & Y_{jl}=\mu (l,d_{j},I_{l};\theta )+\left|\frac{\mu (l,d_{j},I_{l};\theta )}{\mu (L,d_{j},I_{l};\theta )}\right|(s_{j}+I_{l}s_{j}^{*})+(e_{jl}+I_{l}e_{jl}^{*}), \end{array}$$

(5) $$\begin{array}{ll} & \mu (l,d_{j},I_{l};\theta )=\kappa (l,d_{j},I_{l};\theta_{1})\lambda (d_{j},I_{l};\theta_{2}), \end{array}$$

(6) $$\begin{array}{ll} & \kappa (l,d_{j},I_{l};\theta_{1})=\frac{1-\exp(-[k(d_{j})+I_{l}z(d_{j})]t_{l})}{1-\exp(-[k(d_{j})+I_{l}z(d_{j})]t_{L})}, \end{array}$$

(7) $$\begin{array}{ll} & \lambda (d_{j},I_{l};\theta_{2})=(\phi_{0}+I_{l}\delta_{0})+\frac{(\phi_{1}+I_{l}\delta_{1})m_{d_{j}}}{\phi_{2}+I_{l}\delta_{2}+m_{d_{j}}}. \end{array}$$

In the above model, three additional components are introduced to address potential discrepancies between centralized and decentralized data:

1. In the time‐effect model (6), the new parameter z(d) quantifies the magnitude of decentralized shifts in the speed at which the drug takes effect for dose d. A positive value of z(d) indicates that decentralized data suggest a “false” faster onset of the drug effect, that is, an overestimated treatment effect during the initial visits, and vice versa.
2. Similar to the time‐effect model, the inclusion of $\left(I_{l}\delta_{0},I_{l}\delta_{1},I_{l}\delta_{2}\right)$ in (7) distinguishes the decentralized dose‐response model from the centralized model.
3. To account for potential inflation in data variability from decentralized data collection, we introduce two additional residual parameters in (4): $s_{j}^{*}$, representing the additional between‐patient uncertainty, where $s_{j}^{*}∼N(0,\sigma_{s}^{*2})$, and $e_{jl}^{*}$, denoting the additional within‐patient uncertainty, where $e_{jl}^{*}∼N(0,\sigma_{e}^{*2})$ (l=1,…,L).

**FIGURE 1:**
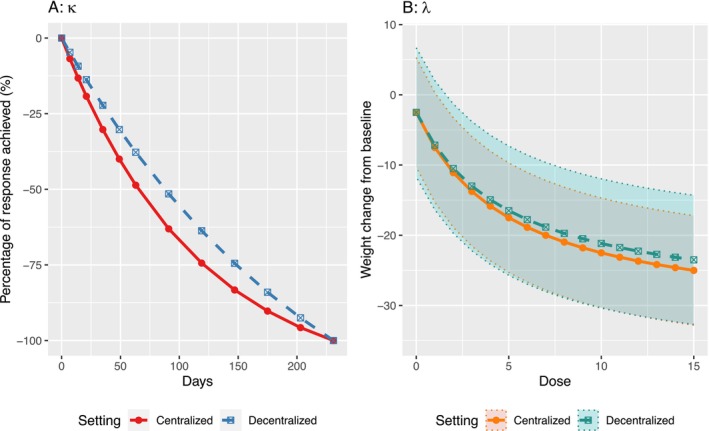
A hypothetical example visualizing increased variability and measurement shifts in the decentralized data. The proposed DCT‐ITP model, based on the settings of the real‐world application (Section 3 and Scenario 3 in Table 1), is used to generate the two panels. Panel (A) depicts the time‐response curve, while Panel (B) shows the dose–response curve at the final visit, with the shaded area representing the 95% confidence region.

Figure 1 visualizes these three components using a hypothetical example. In Panel (A), a shift in the time‐effect curve is shown in the decentralized measurements. In Panel (B), both a shift in the dose‐response curve and increased data variability are observed in the decentralized measurements. In general, when increased variability and measurement shifts are present in the decentralized data, our full model is flexible enough to capture these effects. Moreover, if no bias is introduced by the decentralized collection, the decentralized data can be seamlessly pooled with the centralized data by setting $\delta_{0}=\delta_{1}=\delta_{2}=z(d)=0$.

Remark 1 Although we implement the Emax model to quantify the dose‐response curves, the proposed method, building on the flexibility of the original ITP model, can be readily adapted to accommodate alternative dose‐response models by specifying a tailored function for $\lambda (d_{j},I_{l};\theta_{2})$ in Equation (5). Additional evaluations of different dose‐response models in DCT‐ITP are provided in the Supporting Information.

Remark 2 We posit that the centralized and decentralized dose‐response (or time‐effect) models share the same functional forms but differ in their coefficients. Nonetheless, as evaluated in the sensitivity analysis, this relatively restrictive assumption can still accommodate scenarios in which the centralized and decentralized models follow different functional forms. This robustness is largely attributable to the flexibility of the Emax model and the assumed time‐effect model. Furthermore, the assumptions regarding variance terms in the DCT‐ITP model can be adapted to different scenarios. For instance, if the extent of variability introduced by decentralization is unknown *a priori*, the DCT‐ITP model can be extended to allow independent variance terms, rather than an additive variance structure, for decentralized and centralized measurements.

### 2.3 Bayesian Integrated Estimation

We cast the estimation of the DCT‐ITP model (4) to (7) into the Bayesian framework. In the time‐effect function (6), the original ITP model treats the rate parameters $k_{1}$ to $k_{D}$ for the active doses as independent. However, it is reasonable to assume that different doses of the same treatment follow a similar or exchangeable action pattern. To capture this assumption and enhance the performance of the proposed model, we enable adaptive information sharing across doses by applying Bayesian hierarchical priors [29, 30, 31] for k(1) to k(D), while specifying an independent prior for the placebo‐specific parameter k(0). Specifically, we define the following hierarchical priors for k(1) to k(D):

$$\begin{array}{ll} k(d) & ∼N(\mu_{\kappa },\sigma_{\kappa }^{2}),\;d=1,\ldots ,D,\\ \mu_{\kappa } & ∼N(\theta_{\kappa },\tau_{\kappa }^{2}),\;\sigma_{\kappa }∼\text{HC}(0,\zeta_{\kappa }), \end{array}$$

where $\text{HC}(0,\zeta_{\kappa })$ is the zero‐centered half‐Cauchy distribution with scale parameter $\zeta_{\kappa }$, and $\zeta_{\kappa }$, $\theta_{\kappa }$, and $\tau_{\kappa }^{2}$ are hyperparameters, with $\tau_{\kappa }^{2}$ and $\zeta_{\kappa }$ set to be reasonably large. The variance parameter $\sigma_{\kappa }$ determines the strength of information borrowing. Following Gelman [32], a half‐Cauchy distribution is assigned to $\sigma_{\kappa }$ to facilitate data‐driven information sharing.

For the placebo‐specific parameters k(0), we assign a non‐informative prior instead of including it in the above hierarchy to avoid dilution or over‐borrowing between the placebo and active doses. More specifically, we assign a non‐informative normal prior distribution $N(\mu_{0},\sigma_{0}^{2})$ to the placebo‐specific k(0) with the prior variance $\sigma_{0}^{2}$ set to be reasonably large. Furthermore, if external information is available for the placebo, an informative prior distribution for k(0) can be constructed, for example, using the power prior approach [33], the robust meta‐analytic‐predictive prior approach [34], or other appropriate techniques.

In the time‐effect function (6), we introduce additional terms $I_{l}z(0)$ to $I_{l}z(D)$ to enhance model flexibility and account for biases introduced by decentralization. However, in real‐world scenarios, the presence of such biases is unknown *a priori*. If the location of clinical measurements does not significantly impact the speed of the drug's effect, the term $I_{l}z(d)$ becomes redundant and may even undermine the precision of treatment effect estimation. To address this issue and enable adaptive information sharing across doses, we apply the Bayesian variable selection approach [35, 36, 37] by using hierarchical spike‐and‐slab priors on z(0) to z(D). A discussion of alternative prior choices is provided in the final section of this paper. Specifically, the spike‐and‐slab priors on z(0) to z(D) are formulated as:

(8) $$\begin{array}{ll} z(d) & ∼w_{\kappa }\times \tilde{z}(d)+(1-w_{\kappa })\times \text{Dirac}(0),\;d=0,\ldots ,D,\\ \tilde{z}(d) & ∼N(\mu_{\tilde{z}},\sigma_{\tilde{z}}^{2}),\;d=0,\ldots ,D,\\ \mu_{\tilde{z}} & ∼N(\theta_{\tilde{z}},\tau_{\tilde{z}}^{2}),\;\sigma_{\tilde{z}}∼\text{HC}(0,\zeta_{\tilde{z}}),\;w_{\kappa }∼\text{Ber}(0.5), \end{array}$$

where $w_{\kappa }\in [0,1]$ is the weight of the slab part, Dirac(0) denotes the degenerate prior distribution massed at 0, and $\zeta_{\tilde{z}},\theta_{\tilde{z}},\tau_{\tilde{z}}^{2}$ are hyperparameters, with $\tau_{\tilde{z}}^{2}$ and $\zeta_{\tilde{z}}$ set to be reasonably large. Without specific prior information, we assign $w_{\kappa }$ a neutral Bernoulli prior distribution that takes the value 1 with probability 0.5. Small posterior values of $w_{\kappa }$ indicate that decentralization introduces minimal bias, allowing for greater information sharing between centralized and decentralized measurements. Conversely, large posterior values of $w_{\kappa }$ suggest that the bias from decentralization is nonnegligible. When $w_{\kappa }\neq 0$, it indicates nonnegligible differences between the decentralized and centralized time‐effect functions. In addition, it is worth noting that we assume all doses share the same weight parameter $w_{\kappa }$, implying that the presence of bias in the rate parameters is consistent across dose levels. This assumption reduces the number of parameters and simplifies Bayesian variable selection.

In the Emax model (7), we assign independent non‐informative normal priors for $\phi_{0}$ and $\phi_{1}$. However, the model is sensitive to the prior specification of $\phi_{2}$ due to the small sample size and the limited number of doses in a phase II trial. Furthermore, using a flat prior on certain parameters in nonlinear models can inadvertently induce an informative distribution on the dependent variable, potentially introducing bias [38]. To mitigate this issue, we apply the functional uniform prior on $\phi_{2}$, a prior that generally provides more robust and theoretically sound performance for nonlinear models [39]. In summary, the priors for $\phi_{0},\phi_{1},$ and $\phi_{2}$ are given as follows:

$$\begin{array}{ll} \phi_{h} & ∼N(\mu_{\phi_{h}},\sigma_{\phi_{h}}^{2}),\;h=0,1,\\ p(\phi_{2}) & \propto ℐ(\phi_{2}\in [0,m_{\max}])\int_{0}^{m_{\max}}\frac{x^{2}}{{\left(x+\phi_{2}\right)}^{4}}dx, \end{array}$$

where $\mu_{\phi_{h}},\sigma_{\phi_{h}}^{2}$ (h=0,1) are hyperparameters, ℐ(·) denotes the indicator function, and the support for $\phi_{2}$ is restricted in $\left[0,m_{\max}\right]$. In general, the value of $m_{\max}$ should be carefully chosen. Based on our experience, selecting a relatively large (but not excessively large) value ensures that the support of $\phi_{2}$ is reasonably covered while avoiding extreme values when $m_{\max}\geq m_{D}$. We choose the functional uniform prior for $\phi_{2}$ because it provides a systematic framework for eliciting priors on nonlinear parameters, while also delivering robust estimation performance as demonstrated by Bornkamp [38]. However, based on our evaluation (results not shown), other reasonable priors for $\phi_{2}$ can also be implemented without any deterioration in performance. Similar to the prior on z(d), we specify independent spike‐and‐slab priors for $\delta_{0}$ to $\delta_{2}$:

$$\begin{array}{ll} \delta_{h} & ∼w_{h}\times N(\mu_{h},\sigma_{h}^{2})+(1-w_{h})\times \text{Dirac}(0),\;h=0,1,2,\\ w_{h} & ∼\text{Ber}(0.5),\;h=0,1,2, \end{array}$$

where $\mu_{h}$ and $\sigma_{h}^{2}$ (h=0,1,2) are hyperparameters, with $\sigma_{h}^{2}$ set to be reasonably large.

Lastly, to complete the prior specification of the DCT‐ITP model, we assign non‐informative half‐Cauchy priors on the residual standard deviation parameters $\sigma_{s}$, $\sigma_{s}^{*}$, $\sigma_{e}$, and $\sigma_{e}^{*}$ as

$$\begin{array}{cc} \sigma_{s} & ∼\text{HC}(0,\zeta_{s}),\;\sigma_{s}^{*}∼\text{HC}(0,\zeta_{s}^{*}),\\ \sigma_{e} & ∼\text{HC}(0,\zeta_{e}),\;\sigma_{e}^{*}∼\text{HC}(0,\zeta_{e}^{*}), \end{array}$$

where the hyperparameters $\zeta_{s},\zeta_{s}^{*},\zeta_{e}$ and $\zeta_{e}^{*}$ should take reasonably large values.

### 2.4 Handling Missing Data

Missing data is a common issue in longitudinal studies [40, 41]. Challenges related to longitudinal missing data arise from various sources, such as dropout (i.e., participants leaving the study before completion, resulting in missing observations at later time points), intermittent missingness (i.e., participants missing specific time points but continuing to participate in others), or measurement errors at certain time points. To address these challenges, we propose using BDA to handle longitudinal missing data under the mechanism of missing at random (MAR) [42, 43].

BDA is a Bayesian technique that treats the unobserved outcomes as unknown parameters to be estimated. This approach allows the missing data to be imputed at each step of the Markov Chain Monte Carlo (MCMC) algorithm, facilitating more accurate inference by integrating over the uncertainty associated with the missing values. Assume that the patient outcomes $Y=\{Y_{jl},j=1,\ldots ,J,l=1,\ldots ,L\}$ are decomposed into two parts: the observed data ($Y_{obs}$) and the missing data ($Y_{mis}$). By treating $Y_{mis}$ as unknown parameters, the BDA approach can be described as an iterative process consisting of the imputation step and the updating step:

1. Imputation step: Treat $Y_{mis}$ as unknown parameters and sample each $Y_{jl}^{m}\in Y_{mis}^{m}$ from the posterior predictive distribution conditioned on the observed data and the current posterior sample of the model parameters $\theta^{m}$:
$$\begin{array}{ll} Y_{jl}^{m} & =\mu (l,d_{j},I_{l};\theta^{m})+\left|\frac{\mu (l,d_{j},I_{l};\theta^{m})}{\mu (L,d_{j},I_{l};\theta^{m})}\right|(s_{j}^{m}+I_{l}s_{j}^{*m})\\ & \;+(e_{jl}^{m}+I_{l}e_{jl}^{*m}), \end{array}$$
  where $s_{j}^{m}∼N(0,{\left(\sigma_{s}^{m}\right)}^{2})$, $s_{j}^{*m}∼N(0,{\left(\sigma_{s}^{*m}\right)}^{2})$, $e_{jl}^{m}∼N(0,{\left(\sigma_{e}^{m}\right)}^{2})$ and $e_{jl}^{*m}∼N(0,{\left(\sigma_{e}^{*m}\right)}^{2})$ for l=1,…,L.
2. Updating step: Update the model parameters θ based on the DCT‐ITP model (4) to (7), the prior distributions in Section 2.3, and the dataset $\left\{Y_{obs},Y_{mis}^{m}\right\}$.

In our paper, we adopt the “Just Another Gibbs Sampler (JAGS)” [44] for efficient MCMC sampling and implementing BDA. The JAGS framework can be effectively executed using the R package r2jags [45].

## 3 Trial Application

### 3.1 Trial Configuration

We apply the proposed model to analyze a hypothetical weight‐loss trial, where evidence suggests that outcomes collected through decentralized methods exhibit a reduced treatment effect [26]. To better reflect reality, our hypothetical trial is derived from a real trial by adopting the same data collection design, endpoints, dose‐response estimates, and variability patterns observed in both onsite and offsite assessments. Specifically, we assume that the average weight change from baseline for the maximum dose in the decentralized data is around 2 kg lower than that reported in the onsite data. Additionally, we assume that decentralized measurements lead to an inflation of about 1 unit in both within‐patient and between‐patient standard deviations. Using this information, we generate individual‐level data for a phase II multidose randomized trial with four active doses $(m_{1},\ldots ,m_{4})=$ (1, 5, 10, and 15 mg) and a placebo arm, with n=24 patients in each arm. The Emax model is used to depict the dose‐response relationship by setting $\phi_{0}=-2.5$, $\phi_{1}=-30$, and $\phi_{2}=5$. The bias introduced by decentralized measurements is modeled with $\delta_{0}=0.5$, $\delta_{1}=2$, and $\delta_{2}=0$. The rate parameters for both onsite and decentralized measurements are kept the same, with k(d)=2 and z(d)=0 for d=1,…,D. Besides, the variances are set as $\sigma_{s}^{2}=36$, $\sigma_{e}^{2}=25$, $\sigma_{s^{*}}^{2}=13$, $\sigma_{e^{*}}^{2}=11$, yielding $\sqrt{\sigma_{s}^{2}+\sigma_{s^{*}}^{2}}=\sigma_{s}+1$ and $\sqrt{\sigma_{e}^{2}+\sigma_{e^{*}}^{2}}=\sigma_{e}+1$. For visualization, Figure 1 displays the time‐effect and dose‐response curves for both centralized and decentralized measurements. The trial includes 12 scheduled visits for each patient. We assume Visit 0 is the baseline visit (Day 0), with (post‐treatment) Visits 1 to 3 (inclusive), Visits 4 to 6 (inclusive) biweekly, and Visits 7 to 12 (inclusive) every four weeks. Due to individual circumstances, the actual timing of each visit may vary. For data generation, we assume an allowable visit window of [−3, 3] days from the scheduled visit day. This visit schedule is also illustrated in Figure S1 in the Supporting Information.

We generate patient dropouts and intermittent missing measurements, with an average missing rate of 20% across all visits, encompassing both decentralized and onsite measurements. During (post‐treatment) Visit 1 through the last Visit 12, we first generate patient dropouts under the mechanism of MAR by assuming

$$\begin{array}{cc} & \text{logit}(\mathrm{Pr}(\text{Patient dropout at}\;t_{l}))\\ & \;=\alpha_{0}+\alpha_{1}Y_{l-1}+\alpha_{2}\log(t_{l}),\;l=1,\ldots ,12, \end{array}$$

where $\alpha_{0}=-5.05$, $\alpha_{1}=-0.02$, and $\alpha_{2}=0.05$, yielding an accumulated dropout rate of 10% at the end of the trial. Consequently, patient dropouts at a specific visit l depend on the outcome of the previous visit $Y_{l-1}$ as well as the time $t_{l}$. Similarly, if a patient does not drop out, we assume that the probability of intermittent missingness at a specific visit l (l=1,…,12) as,

(9) $$\begin{array}{cc} & \text{logit}(\mathrm{Pr}(Y_{l}\;\;\mathrm{is}\;\text{missing}|\text{Active patient}))\\ & \;=(\beta_{0}+I_{l}\delta_{\beta_{0}})+(\beta_{1}+I_{l}\delta_{\beta_{1}})Y_{l-1}+\beta_{2}\log(t_{l}), \end{array}$$

where $\beta_{0}=-3.25$, $\delta_{\beta_{0}}=-0.3$, $\beta_{1}=-0.15$, $\delta_{\beta_{1}}=0$, $\beta_{2}=0.05$, yielding an average intermittent missing rate of 16% throughout the trial. In Figure S2 of the Supporting Information, we display the accumulated dropout rates and intermittent missingness rates over time.

### 3.2 Model Evaluation

We compare the proposed DCT‐ITP model with the conventional ITP model across three different settings. In the first setting, we fit the ITP model using only centralized measurements (O‐ITP). In the second setting, we apply the ITP model without distinguishing between centralized and decentralized data (M‐ITP), thereby ignoring any potential biases and uncertainties introduced by decentralization. In the third setting, we consider a traditional trial where all 12 post‐treatment measurements are measured at traditional clinical trial sites (T‐ITP). Since the T‐ITP approach is expected to yield the best performance, we use it as a benchmark for comparison. To ensure comparable missing rates, we apply the same missing data generation mechanism described earlier to all four approaches. To implement the ITP models, we set the following hyperparameters: $\mu_{\phi_{0}}=-2$, $\mu_{\phi_{1}}=-30$, $\sigma_{\phi_{0}}^{2}=\sigma_{\phi_{1}}^{2}=20$, $m_{\max}=20$, $k(0)∼N(2,2^{2})$, $\theta_{\kappa }=\tau_{\kappa }=\zeta_{\kappa }=2$; $\mu_{h}=0$, $\sigma_{h}=4$, for h=0,1,2; $\theta_{\tilde{z}}=0$, $\tau_{\tilde{z}}=\zeta_{\tilde{z}}=1$; $\zeta_{s}=\zeta_{s}^{*}=\zeta_{e}=\zeta_{e}^{*}=2$.

In our analysis, we focus on the following four parameters of clinical interest:

- AUC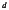: The area under the time‐effect curve (AUC) under all scheduled post‐treatment visits, AUC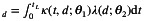, for d=0,…,D.
- ΔAUC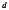: The difference in AUC between an active dose d>0 and the placebo d=0, $\Delta {\text{AUC}}_{d}={\text{AUC}}_{d}-{\text{AUC}}_{0},$ for d=1,…,D.
- $\mu_{d}$: The mean response at the final visit $t_{L}$, for d=0,…,D.
- $\Delta \mu_{d}$: The difference in mean response between an active dose and the placebo, $\Delta \mu_{d}=\mu_{d}-\mu_{0},$ for d=1,…,D.

**FIGURE 2:**
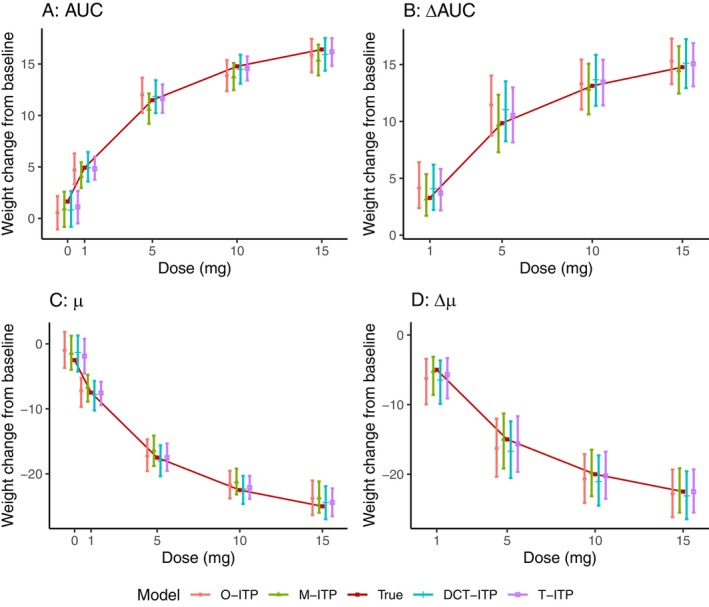
Posterior mean estimates of the area under the time‐effect curve (AUC), the difference in AUC between an active dose and the placebo (ΔAUC), the mean response at the final visit ($\mu_{d}$), and the difference in mean response between an active dose and the placebo ($\Delta \mu_{d}$), along with the associated 95% credible interval based on the four different ITP models for the real‐world application in Section 3.

Figure 2 summarizes the posterior mean estimates and 95% credible intervals for the above four quantities, based on the four ITP approaches. Compared to the proposed DCT‐ITP model, the O‐ITP approach produces similar point estimates but with wider credible intervals, indicating lower efficiency. In contrast, the M‐ITP model yields narrower credible intervals than the DCT‐ITP model, but these intervals sometimes fail to cover the true values. Among the four approaches, T‐ITP performs best, with minimal bias and uncertainty. Nevertheless, the DCT‐ITP model performs very similarly to T‐ITP overall. This indicates that a decentralized trial using our DCT‐ITP approach can achieve a similar level of accuracy and efficiency as a traditional trial with the conventional ITP model.

## 4 Simulation Studies

We conduct simulation studies to further assess the performance of the proposed DCT‐ITP model using eight various scenarios. As shown in Table 1, these scenarios cover a wide range of potential scenarios relevant to the phase II dose‐ranging trials and various impacts of decentralization. Scenarios 1‐6 use the true DCT‐ITP parameterization to generate the data, while Scenarios 7 and 8 represent cases where the dose‐response model is misspecified. Scenario 1 represents the case where onsite and decentralized measurements produce identical results. Scenario 2 represents unbiased decentralized measurements with increased data variability. Scenario 3 corresponds to the motivating example, where both biases and inflated variability are expected due to decentralization. Scenarios 4 to 6 illustrate various potential differences between onsite and decentralized measurements. Scenario 7 assumes both the onsite and decentralized dose‐response curves follow a quadratic model, but with different parameter values. Scenario 8 assumes the onsite curve follows a quadratic model, while the decentralized curve follows a linear pattern. In all scenarios, we assume that the weight change at the final visit (i.e., $\mu_{d}$) ranged from −3 to −25 kg, consistent with clinical trial results reported for approved glucagon‐like peptide‐1 (GLP‐1) receptor agonists. Furthermore, based on findings from a recent weight‐loss study [10], which reported an average bias of −3 kg for off‐site measurements, we assume that decentralization could introduce an absolute bias ranging from 0–6 kg. The true values of $\mu_{d}$, along with the AUC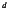 values for each dose under each scenario, are presented in Tables S2 and S3 of the Supporting Information, respectively. The missing data generation follows the procedure outlined in Section 3.1, maintaining an average missing rate of 20% across all visits for each scenario. The corresponding missing model parameters are detailed in Table S1 of the Supporting Information.

**TABLE 1:** True parameter settings for Scenarios 1 to 8.

Scenario	$\phi_{0}\;(\delta_{0})$	$\phi_{1}\;(\delta_{1})$	$\phi_{2}\;(\delta_{2})$	k(d)(z(d))	$\sigma_{s}^{2}\;({\left(\sigma_{s}^{*}\right)}^{2})$	$\sigma_{e}^{2}\;({\left(\sigma_{e}^{*}\right)}^{2})$
1	−2.5 (0)	−30 (0)	5 (0)	2 (0)	36 (0)	25 (0)
2	−2.5 (0)	−30 (0)	5 (0)	2 (0)	36 (13)	25 (11)
3	−2.5 (0.5)	−30 (2)	5 (0)	2 (0)	36 (13)	25 (11)
4	−2.5 (−0.5)	−30 (−2)	5 (0)	2 (0)	36 (13)	25 (11)
5	−2.5 (0)	−25.5 (5.5)	2 (0.5)	1 (−0.5)	64 (36)	36 (28)
6	−2.5 (−0.5)	−28 (−2)	6 (−0.5)	1 (0∼2)^a^	64 (36)	36 (28)
7	Onsite: $\lambda =-1-2.2m_{d}+0.05m_{d}^{2}$			2 (1)	81 (40)	64 (36)
	Decentralized: $\lambda =-1-2m_{d}+0.037m_{d}^{2}$					
8	Onsite: $\lambda =-1-2.1m_{d}+0.05m_{d}^{2}$			2 (0)	25 (11)	36 (13)
	Decentralized: $\lambda =-4-1.4m_{d}$					

^a^ In Scenario 6, z(d) varies by dose, that is, z(0)=0,z(1)=0.5z(2)=1,z(3)=1.5,z(4)=2.

We apply the four ITP models described in Section 3.2, using the same prior specifications, to each simulation scenario, with results obtained from 1000 replications. For each parameter of interest $\Theta_{d}\in$(${\text{AUC}}_{d}$, $\Delta {\text{AUC}}_{d}$, $\mu_{d}$, $\Delta \mu_{d}$), we report the average bias (AB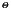), the average root mean square error (ARMSE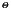), the average coverage probability (ACP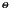) of pointwise 95% credible intervals (CIs), and the average length (AL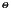) of the 95% CIs, averaged across different doses:

(10) $$\begin{array}{cc} {\text{AB}}_{\Theta } & =\frac{1}{\left|𝒟\right|}\underset{d\in 𝒟}{\sum }\;\overset{N}{\underset{n=1}{\sum }}\frac{1}{N}\left({\hat{\Theta }}_{d,n}-\Theta_{d}\right),\\ {\text{ARMSE}}_{\Theta } & =\frac{1}{\left|𝒟\right|}\underset{d\in 𝒟}{\sum }\sqrt{\frac{1}{N}\overset{N}{\underset{n=1}{\sum }}{\left({\hat{\Theta }}_{d,n}-\Theta_{d}\right)}^{2}},\\ {\text{ACP}}_{\Theta } & =\frac{1}{\left|𝒟\right|}\underset{d\in 𝒟}{\sum }\;\overset{N}{\underset{n=1}{\sum }}\frac{I(\Theta_{d}\in ({\hat{\Theta }}_{d,n}^{l},{\hat{\Theta }}_{d,n}^{u})}{N},\\ {\text{AL}}_{\Theta } & =\frac{1}{\left|𝒟\right|}\underset{d\in 𝒟}{\sum }\;\overset{N}{\underset{n=1}{\sum }}\frac{\left({\hat{\Theta }}_{d,n}^{u}-{\hat{\Theta }}_{d,n}^{l}\right)}{N}, \end{array}$$

where |𝒟| denotes the number of doses in the dose set 𝒟, ${\hat{\Theta }}_{d,n}$, ${\hat{\Theta }}_{d,n}^{l}$ and ${\hat{\Theta }}_{d,n}^{u}$ are the posterior mean estimate, the 2.5% and 97.5% posterior quantile estimates of $\Theta_{d}$ in the nth replicated trial, respectively. In our simulation, the target dose set 𝒟 corresponds to (0, 1, 5, 10, and 15 mg) for AUC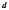, (1, 5, 10, and 15 mg) for $\Delta {\text{AUC}}_{d}$, (0, 1, 2, …, 15 mg) for $\mu_{d}$, and (1, 2, …, 15 mg) for $\Delta \mu_{d}$, respectively.

We employ the r2jags package [45] with an initial burn‐in of 5000 iterations, followed by 10 000 sampling iterations with a thinning interval of 2 to perform MCMC sampling. On a MacBook Pro 14 (Apple M2 Pro chip with 12‐core CPU), each simulation replication requires approximately 12.9 s, yielding a total runtime of about 3.6 h for 1000 replications. This computation time could be substantially reduced through parallel computing. To assess convergence, we apply Geweke's diagnostic by comparing the first 10% and the last 50% of the Markov chain. Across 1000 replications, the diagnostic consistently produces p‐values above the 0.05 threshold, providing strong evidence of satisfactory convergence.

**FIGURE 3:**
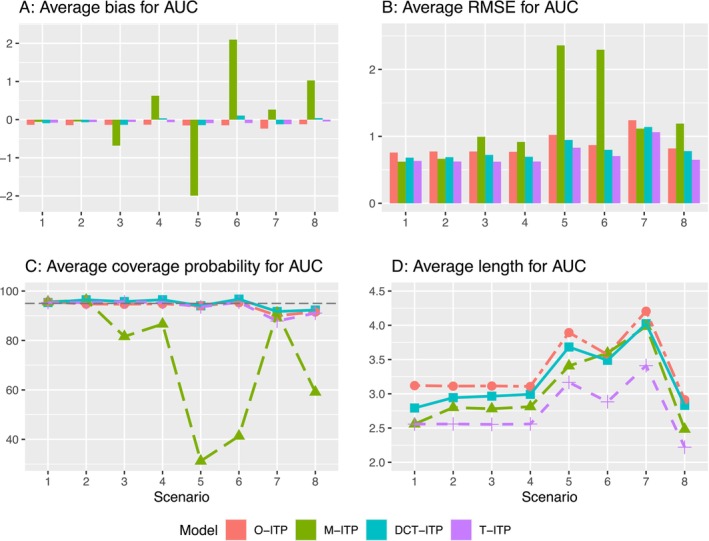
Average bias, average root mean square error (RMSE), and average coverage probability and length of pointwise 95% credible intervals for the estimate of the area under the time‐response curve (AUC) using the four integrated two‐component prediction (ITP) models under Scenarios 1 to 8.

**FIGURE 4:**
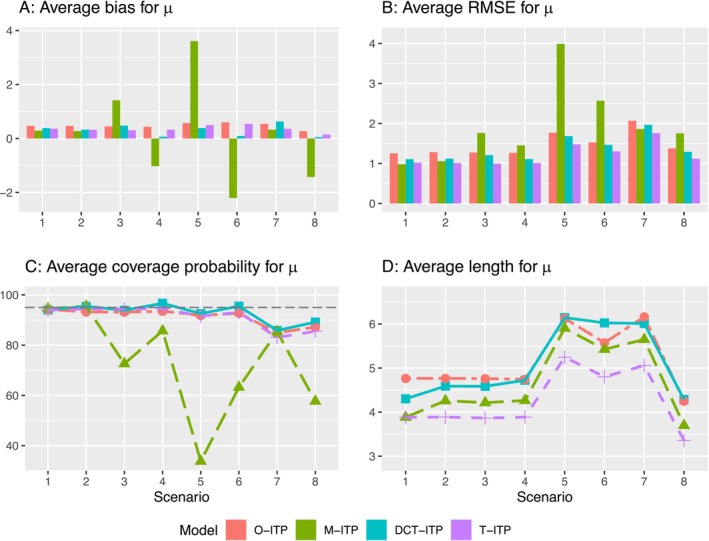
Average bias, average root mean square error (RMSE), and average coverage probability and length of pointwise 95% credible intervals for the estimate of the mean response at the final visit (μ) using the four integrated two‐component prediction (ITP) models under Scenarios 1 to 8.

The results for ${\text{AUC}}_{d}$ and $\mu_{d}$ are shown in Figures 3 and 4, while the results for $\Delta {\text{AUC}}_{d}$ and $\Delta \mu_{d}$ are presented in Figures S3 and S4 in the Supporting Information. In Scenarios 1 and 2, where decentralization does not introduce biases, the M‐ITP and proposed DCT‐ITP models perform comparably. However, the O‐ITP approach exhibits greater bias, higher ARMSE, and wider 95% CIs, as it relies solely on centralized measurements, which are taken at sparse time points. In Scenarios 3 to 6, where biases are present in decentralized measurements, the M‐ITP approach demonstrates significant shortcomings, leading to large biases, inflated ARMSE, and limited ACP. In contrast, our DCT‐ITP approach adaptively detects and corrects for decentralization bias, resulting in much better performance than M‐ITP. In Scenario 7, despite the misspecification of the dose‐response model, DCT‐ITP yields relatively robust results due to the similarities between the quadratic and Emax models. In Scenario 8, approximating the linear dose‐response relationship using the Emax model proves more challenging, resulting in limited performance across all methods. Nevertheless, the proposed model still outperforms both the M‐ITP and O‐ITP approaches. Overall, the O‐ITP approach performs relatively robustly across various scenarios, but its reliance on centralized measurements alone leads to information loss. The M‐ITP approach is highly sensitive to discrepancies between onsite and decentralized measurements, with larger differences leading to worse performance. Compared to these two naïve approaches, the proposed model provides a more effective method for analyzing longitudinal dose‐response data in DCTs. Additionally, the T‐ITP approach generally performs best across all scenarios, as expected. However, our DCT‐ITP approach offers similarly competitive performance. It is important to highlight the intrinsic limitations of traditional clinical trials, including participant inconvenience, limited diversity in the participant pool, and additional operational challenges for investigators.

In Section S2 of the Supporting Information, we present additional simulation results to further illustrate the necessity of the complex modeling in DCT‐ITP. Specifically, we compare the proposed DCT‐ITP model with two simplified variations while keeping all other parameters and priors unchanged: (i) setting $w_{h}=w_{\kappa }=1$ to deactivate Bayesian variable selection and (ii) specifying $k(d)∼N(2,2^{2})$ and $\tilde{z}(d)∼N(0,1)$ to remove hierarchical borrowing across the time‐effect coefficients k(1) through k(D) and $\tilde{z}(0)$ through $\tilde{z}(D)$. Under the same eight simulation scenarios, the proposed DCT‐ITP consistently outperforms these simplified alternatives in terms of ARMSE and AL, highlighting the importance of the complex modeling components.

Furthermore, to assess the versatility of DCT‐ITP in accommodating different dose‐response models $\lambda (d_{j},I_{l};\theta_{2})$, Section S3 of the Supporting Information evaluates the DCT‐ITP model under both linear and quadratic dose‐response specifications and compares its performance with the O‐ITP, M‐ITP, and T‐ITP counterparts. Overall, the simulation results show that, across different parameterizations of $\lambda (d_{j},I_{l};\theta_{2})$, the proposed DCT‐ITP model consistently demonstrates estimation advantages over O‐ITP and M‐ITP.

## 5 Sensitivity Analyses

We investigate the impact of different hyperparameter values, trial settings, and model configurations on the performance of the proposed DCT‐ITP method through a series of sensitivity analyses, with detailed results provided in the Supporting Information. In each of the following sensitivity analyses, unless otherwise specified, the prior distributions are the same as those described in Section 3.2.

- Prior weights $w_{\kappa }$ and $w_{h}$ (h=0,1,2): In addition to the neutral setting where both $w_{\kappa }$ and $w_{h}$ follow a Ber(0.5) distribution, we also consider Ber(0.75) and Ber(0.25) to respectively discount or promote information sharing between centralized and decentralized measurements. As expected, Figure S7 shows that the prior weights have a relatively small but interpretable impact on estimation performance: with smaller prior weights, the DCT‐ITP tends to shrink decentralized measurements more strongly toward centralized measurements, and vice versa. Consequently, DCT‐ITP with smaller prior weights performs better when there is little or no deviation between centralized and decentralized measurements (i.e., Scenarios 1 and 2), whereas larger prior weights perform better when some deviation exists. Overall, the neutral prior setting with a Ber(0.5) distribution provides a balanced trade‐off between efficiency and robustness, and can be considered a reasonable default when no information about decentralization bias is available *a priori*.
- Variance of the slab component: In the spike‐and‐slab prior, the slab variance $\sigma_{h}^{2}$ governs the degree of information borrowing between centralized and decentralized measurements, with smaller values of $\sigma_{h}^{2}$ leading to greater borrowing [46]. In Figure S8, we compare the performance of DCT‐ITP with $\sigma_{h}^{2}=2^{2},4^{2},$ and $6^{2}$. The results show that the ARMSE, ACP, and AL remain visually identical across the three prior variance settings, although smaller values of $\sigma_{h}^{2}$ yield larger estimation bias, particularly in Scenarios 3–8.
- Prior scales ($\zeta_{\kappa }$ and $\zeta_{\tilde{z}}$) on the heterogeneity parameters: These scale parameters control the degree of shrinkage across dose levels in the hierarchical prior for the time‐effect coefficients in Equation (6). In addition, $\zeta_{\tilde{z}}$ also determines the variance of the slab component in Equation (8). To assess their impact, we consider two alternative configurations: ($\zeta_{\kappa }$, $\zeta_{\tilde{z}}$) = (1, 0.5) and (3, 2). As shown in Figure S9, DCT‐ITP remains robust across all considered prior scale settings, with only minimal variation in performance.
- Sample size: We also consider two additional sample sizes: 80 and 200. The results in Figure S10 indicate that increasing the sample size improves the performance of the proposed method, with larger sample sizes yielding better outcomes.
- Visit schema: We evaluate the performance of the proposed method across different measurement schemas. Specifically, we simulate a trial with (1) a total of 8 visits, including 5 decentralized visits; (2) 12 visits, including 4 decentralized visits; and (3) 15 visits, including 9 decentralized visits. Schemas (1) and (3) maintain a similar proportion of decentralized visits but differ in the total number of visits, while schema (2) represents a lower proportion of decentralized visits. The three additional schemas are illustrated in Figure S1B–D in the Supporting Information, respectively. The results in Figure S11 show that increasing the total number of visits and reducing the degree of decentralization lead to improved performance.
- Missing data: We also examine three different missing data settings. In the first two settings, we adjust the parameters $\alpha_{0}$ and $\alpha_{1}$ to achieve average missing rates of approximately 10% and 30% across all visits. In the third setting, we consider a combination of intermittent missing completely at random (MCAR), intermittent MAR, and random dropout, each with an average rate of approximately 10%, resulting in a total average missing rate of 30%. The missing data generation mechanism for the third setting is detailed in Section S1 of the Supporting Information. The specifications for these three settings are summarized in Table S1 in the Supporting Information. According to Figure S12, the results indicate that the performance of the proposed model improves as the missing rate decreases.
- Model violation: The DCT‐ITP model assumes that the centralized and decentralized dose–response (as well as time‐effect) models share the same functional forms but may differ in their coefficients. To evaluate the performance of DCT‐ITP when this assumption is violated, we construct 18 additional scenarios in Section S4 of the Supporting Information by allowing the centralized and decentralized models to follow different functional forms. As shown in Figure S13, across these 18 scenarios, the DCT‐ITP model outperforms both the O‐ITP and M‐ITP models in terms of estimation efficiency and accuracy, consistent with the conclusions drawn in Section 4.

## 6 Concluding Remarks

We introduce a novel multilevel Bayesian modeling and learning approach for evaluating longitudinal data from decentralized multidose randomized trials. Our DCT‐ITP model builds upon the ITP model, enhancing it by integrating information across doses and between centralized and decentralized measurements. Given that the presence and magnitude of bias due to decentralization are typically unknown before the trial begins, we employ a Bayesian spike‐and‐slab prior technique on the relevant parameters to enable data‐adaptive information integration. We evaluate the performance of the proposed DCT‐ITP model using the endpoint of change in body weight, with parameters and settings derived from a real clinical trial where body weights are measured through both centralized and decentralized methods. Through extensive numerical studies, the proposed model demonstrates desirable precision and stability in estimation. Sensitivity analyses further confirm the robustness of the design under various trial and prior settings. Although patient covariates are not included in the DCT‐ITP model, incorporating them as an additive term would be straightforward.

In this paper, we choose the spike‐and‐slab prior to adaptively assess potential biases introduced by decentralization, given its wide adoption in clinical trial applications (e.g., historical borrowing), ease of hyperparameter elicitation, and straightforward interpretation. Nevertheless, with appropriate specification, other Bayesian variable selection priors, such as the horseshoe prior [47], could also be applied to model z(d) and $\delta_{h}$. As a preliminary investigation, we have additionally implemented the horseshoe prior in our simulation scenarios. Specifically, the horseshoe priors for z(d) and $\delta_{h}$ are given as follows:

$$\begin{array}{ll} & z(d)∼N(0,\lambda_{d}^{2}\tau_{z}^{2}),\;\lambda_{d}∼\text{HC}(0,\xi_{\lambda }),\;d=0,\ldots ,D,\\ & \delta_{h}∼N(0,\lambda_{h}^{2}\tau_{\delta }^{2}),\;\lambda_{h}∼\text{HC}(0,\xi_{h}),\;h=0,1,2,\\ & \tau_{z}∼\text{HC}(0,\xi_{z}),\;\tau_{\delta }∼\text{HC}(0,\xi_{\delta }), \end{array}$$

where the hyperparameters $\xi_{\lambda },\xi_{h},\xi_{z},\xi_{\delta }$ are all set to 1 in this simulation, ensuring that approximately 50% of the prior densities of z(d) and $\delta_{h}$ are concentrated near zero (i.e., comparable to our setting of the spike‐and‐slab priors). As shown in Figure S14, overall, the spike‐and‐slab and horseshoe priors produced very similar average RMSE values. However, the spike‐and‐slab prior generally yielded slightly smaller estimation bias across most scenarios and a narrower 95% credible interval in some cases. This finding is consistent with a recent comparative study by [48] on historical borrowing, which concluded that the spike‐and‐slab prior achieved the best overall performance when borrowing from heterogeneous historical controls. Nonetheless, extensive evaluation of different Bayesian variable selection priors is warranted in the context of DCTs.

We focus on addressing the increased variability and potential bias introduced by decentralization. However, additional challenges may arise in decentralized trials. A significant challenge is participant engagement, as geographical isolation can affect adherence and increase missing data. At present, the proposed DCT‐ITP model can only accommodate MAR scenarios. Further methodological development is warranted to extend the approach to settings with missing‐not‐at‐random data. From a practical standpoint, mitigating the risk of missing data requires anticipating potential missingness scenarios and carefully planning the frequency and timing of decentralized activities before trial initiation. During the trial, providing adequate support and maintaining consistent communication with participants are crucial for promoting engagement and minimizing missing data. Practical challenges such as technology infrastructure, data integration, security, logistics, and regulatory compliance are also critical considerations. Addressing these challenges will be an important area for future research.

Another unresolved question concerns the appropriate definition of the estimand for DCTs. In this paper, we treat the estimand as the parameter of interest, assuming that all visits are centralized, following the traditional clinical trial approach, which remains the gold standard for treatment evaluation. However, this assumption may not always hold. For example, decentralized trials may offer a less biased evaluation in clinical trials for sleep disorders. As far as we know, only one paper by [49] has explored the estimand framework for decentralized trials. However, achieving consensus on the appropriate estimand among various stakeholders in decentralized trials remains a long‐term goal.

## Funding

The authors have nothing to report.

## Conflicts of Interest

The authors declare no conflicts of interest.

## Supporting information




**Data S1**: sim70338‐sup‐0001‐Supplementary Materials.pdf.

## Data Availability

The authors have nothing to report.
