# Supplementary material for: Bayesian Integrated Learning of Longitudinal Dose‐Response Relationships via Decentralized Clinical Trials
Source: Stat Med. 2025 Dec 3;44(28-30):e70338. doi: 10.1002/sim.70338 (PMC12675892; doi:10.1002/sim.70338)
Supplement: Supplementary file 1 — Data S1: sim70338‐sup‐0001‐Supplementary Materials.pdf. [file SIM-44-0-s001.zip › sim70338-sup-0001-SupplementaryMaterials.pdf]

# Supplementary Materials for "Bayesian Integrated Learning of Longitudinal Dose–Response Relationships via Decentralized Clinical Trials"

**Jingyi Zhang[1], Tuo Wang[2], Yongming Qu[2], Fangrong Yan[1], Suyu Liu[3], and Ruitao Lin[3*]**

[1] Research Center of Biostatistics and Computational Pharmacy, China Pharmaceutical University, Nanjing, China

[2] Department of Global Statistical Sciences, Eli Lilly and Company, Indianapolis, Indiana, USA

[3] Department of Biostatistics, The University of Texas MD Anderson Cancer Center Houston, Texas 77030, U.S.A.

[*]Corresponding authors: rlin@mdanderson.org

## S1 Missing data generation for sensitivity analysis

In this paper, we assess the performance of the proposed DCT-ITP model under various missing data conditions. In settings (i) and (iii), we apply the missing data generation method described in Section 3.1 of the main paper, setting the overall missing rate to either 10% or 30%. Setting (ii) corresponds to the main simulation study, with an overall missing rate of 20%.

In setting (iv), we incorporate patient dropout under the MAR mechanism, resulting in a mixture of intermittent MCAR, intermittent MAR, and dropout at random, with an average missing rate of 30% across all visits. From post-treatment Visit 1 through the last Visit 12, intermittent missing data under the MAR mechanism are generated following the model:

$$\text{logit}(\Pr(Y_l \text{ is missing} \mid \text{Active patient})) = (\beta_0 + I_l\delta_{\beta_0}) + \beta_1 Y_{l-1}, \quad l = 1, \cdots, 12, \quad \text{(S1.1)}$$

where the parameter settings used in this model are detailed in Table S1 (setting (ii)) to yield an average missing rate of 10% across scenarios. Additionally, a patient measurement may also be missing under the MCAR mechanism, with an MCAR probability equal to the MAR rate for Visits 1–12. Consequently, the average overall missing rate throughout the trial is 20%, with half of the data missing via MCAR and the other half via MAR.

For Visit 1 through Visit 12, patient dropouts are generated according to the following model:

$$\text{logit}(\Pr(\text{Patient dropout at } t_l)) = (\alpha_0 + I_l \delta_{\alpha_0}) + \alpha_1 Y_{l-1}, \quad l = 2, \cdots, 12.$$

The parameters are set to yield an accumulated dropout rate of 20% and an average intermittent MAR rate of 12% throughout the trial. The MCAR probability is set as the same as the MAR rate for each visit. As a result, the average missing rate is 30% across all visits. The parameter settings for the missing data generation models are presented in Table S1, and the simulation results across various missing data types are illustrated in Figure S12.

# S2 Comparison to simplified models

To evaluate the necessity of the complex modeling in DCT-ITP, we compared the proposed DCT-ITP model with two simplified variations while keeping all other parameters and priors unchanged:

(1) Model without Bayesian variable selection: We set $w_h = w_\kappa = 1$ (i.e., retaining only the slab component of the spike-and-slab prior) to deactivate Bayesian variable selection.

(2) Model without hierarchical borrowing across time-effect coefficients: We take $k(d) \sim \text{N}(2, 2^2)$ for $d = 1, \ldots, D$ and $\tilde{z}(d) \sim \text{N}(0, 1)$ for $d = 0, 1, \ldots, D$, such that the time-effect coefficients are independently modeled across doses without information sharing.

As shown in Figure S5, under the same eight simulation scenarios considered in Section 4, the proposed DCT-ITP model achives a similar average bias and coverage probability as

the simplified models, but yields smaller average RMSE and narrower 95% credible intervals in most scenarios. These results highlight the efficiency gains obtained by incorporating the complex modeling components.

# S3 Accommodation of different dose–response models

To demonstrate the versatility of DCT-ITP in accommodating different dose-response models, we parameterize $\lambda(d_j, I_l; \boldsymbol{\theta}_2)$ in equation (5) using linear and quadratic functions:

$$\text{Linear}: \lambda_l(d_j, I_l; \boldsymbol{\theta}_{l,2}) = (\phi_{l,0} + I_l\delta_{l,0}) + (\phi_{l,1} + I_l\delta_{l,1})m_{d_j},$$

$$\text{Quadratic}: \lambda_q(d_j, I_l; \boldsymbol{\theta}_{q,2}) = (\phi_{q,0} + I_l\delta_{q,0}) + (\phi_{q,1} + I_l\delta_{q,1})m_{d_j} + (\phi_{q,2} + I_l\delta_{q,2})m_{d_j}^2,$$

where $\boldsymbol{\theta}_{l,2} = \{\phi_{l,0}, \phi_{l,1}, \delta_{l,0}, \delta_{l,1}\}$ and $\boldsymbol{\theta}_{q,2} = \{\phi_{q,0}, \phi_{q,1}, \phi_{q,2}, \delta_{q,0}, \delta_{q,1}, \delta_{q,2}\}$ denote the corresponding vectors of unknown parameters. For the linear model, the corresponding prior distributions are set as $\phi_{l,0} \sim \mathrm{N}(-2.5, 2^2)$, $\phi_{l,1} \sim \mathrm{N}(-2, 2^2)$, $\delta_{l,i} \sim w_{l,i} \times \mathrm{N}(0, 4^2) + (1 - w_{l,i}) \times \mathrm{Dirac}(0), w_{l,i} \sim \mathrm{Ber}(0.5),\ i = 0, 1$. For the quadratic model, the corresponding priors are set as: $\phi_{q,0} \sim \mathrm{N}(0, 2^2)$, $\phi_{q,1} \sim \mathrm{N}(-1, 2^2)$, $\phi_{q,2} \sim \mathrm{N}(-0.05, 1)$, $\delta_{q,i} \sim w_{q,i} \times \mathrm{N}(0, 4^2) + (1 - w_{q,i}) \times \mathrm{Dirac}(0), w_{q,i} \sim \mathrm{Ber}(0.5),\ i = 0, 1, 2$. The prior distributions for the remaining parameters remain unchanged from those specified in Section 3.1.

To evaluate the performance of DCT-ITP under different dose-response model parameterizations, we additionally consider a total of 12 scenarios (= 6 + 6), as summarized in Tables S4 and S5. Based on 1,000 simulation replications per scenario, Figure S6 demonstrates that the proposed DCT-ITP model consistently outperforms the O-ITP model in estimation efficiency and the M-ITP model in estimation accuracy.

# S4 Sensitivity analysis under model violations

We evaluate the performance of the proposed DCT-ITP model under conditions where deviations in decentralized measurements violate the model assumptions. As summarized in

Table S6, we consider 16 additional data-generation scenarios in which the centralized and decentralized dose-response (or time-effect) models follow different functional forms.

In Scenarios S3.1–S3.6, the centralized and decentralized time-effect models share the same functional form, whereas the centralized and decentralized dose-response models differ. Specifically, the centralized dose-response model follows the Emax function with parameter settings identical to those in Scenario 1 of Table 1, while the decentralized dose-response model adopts a quadratic form:

$$\lambda_q(d_j; \boldsymbol{\theta}_{q,2}) = \phi_{q,0} + \phi_{q,1} m_{d_j} + \phi_{q,2} m_{d_j}^2,$$

where $\boldsymbol{\theta}_{q,2} = \{\phi_{q,0}, \phi_{q,1}, \phi_{q,2}\}$. Among these, Scenarios S3.1 and S3.2 reflect small decentralization bias relative to the centralized dose-response curve, S3.3 and S3.5 reflect moderate bias, and S3.4 and S3.6 reflect large bias.

In Scenarios S3.7–S3.12, the centralized and decentralized dose-response models share the same functional form, whereas the centralized and decentralized time-effect models differ. Specifically, the centralized time-effect model follows equation (6) with parameter settings identical to those in Scenario 1 of Table 1, while the decentralized time-effect model adopts the following form:

$$\kappa(t_l; a_1, a_2) = \frac{\Phi(\frac{t_l - a_1}{a_2})}{\Phi(\frac{t_L - a_1}{a_2})}, l = 1, \cdots, L,$$

where $\Phi(\cdot)$ denotes the standard normal cumulative distribution function. Among these, Scenarios S3.7 and S3.8 represent small bias relative to the centralized time-effect curve, S3.9 and S3.11 represent moderate bias, and S3.10 and S3.12 represent large bias.

Furthermore, Scenarios S3.13–S3.18 are designed to evaluate the performance of the proposed DCT-ITP model when both the dose-response and time-effect models follow different functional forms. Specifically, these scenarios are defined as paired combinations of the corresponding dose-response and time-effect deviations: S3.13 combines S3.1 and S3.7, S3.14 combines S3.2 and S3.8, $\cdots$, and S3.18 combines S3.6 and S3.12.

As shown in Figure S13, across all 18 prespecified scenarios, the DCT-ITP model consistently outperforms both O-ITP and M-ITP in estimating the AUC, yielding lower bias and ARMSE and thus higher accuracy and precision. Moreover, both the credible intervals of T-ITP and DCT-ITP achieve coverage probabilities close to the nominal 95% level while maintaining reasonable credible interval lengths. Taken together, these results demonstrate that the DCT-ITP approach delivers robust performance even under model misspecification.

Table S1: Parameter settings for the missing data generation models. Settings (i)–(iii) represents the mixture of intermittent MCAR and intermittent MAR, with the total average missing rate at each visit at about 10%, 20%, and 30%, respectively. Setting (ii) represents the setting that used for generating missing data for the main simulation studies. Setting (iv) represents the mixture of intermittent MCAR, intermittent MAR, and dropout at random, with the total average missing rate of 30%.

| Scenario | Intermittent MAR | | | | | Dropout | | | |
|---|---|---|---|---|---|---|---|---|---|
| | $\beta_0$ | $\delta_{\beta_0}$ | $\beta_1$ | $\delta_{\beta_1}$ | $\beta_2$ | $\alpha_0$ | $\delta_{\alpha_0}$ | $\alpha_1$ | $\alpha_2$ |
| Setting (i): Average missing rate = 10% | | | | | | | | | |
| 1 | -3.05 | -0.45 | -0.08 | 0.01 | | -5.8 | | | |
| 2 | -3.05 | -0.45 | -0.08 | 0.01 | | -5.8 | | | |
| 3 | -3.25 | -0.3 | -0.08 | 0 | | -5.75 | | | |
| 4 | -3.25 | -0.1 | -0.08 | 0.04 | 0.05 | -5.75 | 0 | -0.02 | 0.05 |
| 5 | -3.05 | -0.1 | -0.08 | 0.04 | | -5.7 | | | |
| 6 | -3 | -0.5 | -0.08 | 1.3 | | -5.7 | | | |
| 7 | -3.5 | -0.1 | -0.08 | 0 | | -5.75 | | | |
| 8 | -3.4 | -0.1 | -0.08 | 0 | | -5.78 | | | |
| Setting (ii): Average missing rate = 20% | | | | | | | | | |
| 1 | -3.1 | -0.45 | -0.15 | 0.01 | | -4.6 | | | |
| 2 | -3.1 | -0.45 | -0.15 | 0.01 | | -4.6 | | | |
| 3 | -3.25 | -0.3 | -0.15 | 0 | | -4.6 | | | |
| 4 | -3.23 | -0.1 | -0.15 | 0.04 | 0.05 | -4.62 | 0 | -0.02 | 0.05 |
| 5 | -2.95 | -0.1 | -0.15 | 0.04 | | -4.55 | | | |
| 6 | -3.3 | -0.5 | -0.15 | 1.3 | | -4.6 | | | |
| 7 | -3.15 | -0.45 | -0.15 | 0.01 | | -5.05 | | | |
| 8 | -3 | -0.4 | -0.15 | 0.01 | | -5.05 | | | |
| Setting (iii): Average missing rate = 30% | | | | | | | | | |
| 1 | -3.05 | -0.45 | -0.22 | 0.01 | | -4.6 | | | |
| 2 | -3.05 | -0.45 | -0.22 | 0.01 | | -4.6 | | | |
| 3 | -3.15 | -0.3 | -0.22 | 0 | | -4.6 | | | |
| 4 | -3.25 | -0.1 | -0.22 | 0.04 | 0.05 | -4.62 | 0 | -0.02 | 0.05 |
| 5 | -3.05 | -0.1 | -0.25 | 0.04 | | -4.55 | | | |
| 6 | -2 | -1.3 | -0.24 | 1.3 | | -4.6 | | | |
| 7 | -3.05 | -1 | -0.25 | 0.01 | | -4.6 | | | |
| 8 | -2.8 | -0.9 | -0.25 | 0.01 | | -4.55 | | | |
| Setting (iv): Average missing rate = 30% | | | | | | | | | |
| 1 | -2.52 | 0.1 | -0.6 | 0 | | -4.13 | | | |
| 2 | -2.52 | 0.1 | -0.6 | 0 | | -4.13 | | | |
| 3 | -2.52 | 0.1 | -0.6 | 0 | | -4.13 | | | |
| 4 | -2.56 | 0.1 | -0.6 | 0 | 0 | -4.13 | 0.1 | 0 | 0 |
| 5 | -2.45 | 0.1 | -0.6 | 0 | | -4.10 | | | |
| 6 | -2.56 | 0.1 | -0.6 | 0 | | -4.13 | | | |
| 7 | -2.50 | 0.1 | -0.6 | 0 | | -4.15 | | | |
| 8 | -2.50 | 0.1 | -0.6 | 0 | | -4.00 | | | |

Table S2: True mean response ($\mu_d$) at the final visit through Scenarios 1 to 8, where "dose 0" denotes the placebo arm.

| Scenario | Measurement | Dose (mg) | | | | |
|---|---|---|---|---|---|---|
| | | 0 | 1 | 5 | 10 | 15 |
| 1 | Onsite | -2.5 | -7.5 | -17.5 | -22.5 | -25.0 |
| | Remote | -2.5 | -7.5 | -17.5 | -22.5 | -25.0 |
| 2 | Onsite | -2.5 | -7.5 | -17.5 | -22.5 | -25.0 |
| | Remote | -2.5 | -7.5 | -17.5 | -22.5 | -25.0 |
| 3 | Onsite | -2.5 | -7.5 | -17.5 | -22.5 | -25.0 |
| | Remote | -2.0 | -6.7 | -16.0 | -20.7 | -23.0 |
| 4 | Onsite | -2.5 | -7.5 | -17.5 | -22.5 | -25.0 |
| | Remote | -3.0 | -8.3 | -19.0 | -24.3 | -27.0 |
| 5 | Onsite | -2.5 | -11.0 | -20.7 | -23.8 | -25.0 |
| | Remote | -2.5 | -8.2 | -15.8 | -18.5 | -19.6 |
| 6 | Onsite | -2.5 | -6.5 | -15.2 | -20.0 | -22.5 |
| | Remote | -3.0 | -7.6 | -17.3 | -22.4 | -25.0 |
| 7 | Onsite | -1.0 | -3.2 | -10.8 | -18.0 | -22.8 |
| | Remote | -1.0 | -3.0 | -10.1 | -17.3 | -22.7 |
| 8 | Onsite | -1.0 | -3.1 | -10.2 | -16.8 | -20.7 |
| | Remote | -4.0 | -5.4 | -11.0 | -18.0 | -25.0 |

Table S3: True area under the time-effect curve ($\mathrm{AUC}_d$) through Scenarios 1 to 8, where "dose 0" denotes the placebo arm.

| Scenario | Measurement | Dose (mg) | | | | |
|---|---|---|---|---|---|---|
| | | 0 | 1 | 5 | 10 | 15 |
| 1 | Onsite | 1.6 | 4.9 | 11.5 | 14.8 | 16.4 |
| | Remote | 1.6 | 4.9 | 11.5 | 14.8 | 16.4 |
| 2 | Onsite | 1.6 | 4.9 | 11.5 | 14.8 | 16.4 |
| | Remote | 1.6 | 4.9 | 11.5 | 14.8 | 16.4 |
| 3 | Onsite | 1.6 | 4.9 | 11.5 | 14.8 | 16.4 |
| | Remote | 1.3 | 4.4 | 10.5 | 13.6 | 15.1 |
| 4 | Onsite | 1.6 | 4.9 | 11.5 | 14.8 | 16.4 |
| | Remote | 2.0 | 5.5 | 12.5 | 16.0 | 17.7 |
| 5 | Onsite | 1.5 | 6.4 | 12.1 | 13.8 | 14.5 |
| | Remote | 1.4 | 4.5 | 8.6 | 10.0 | 10.6 |
| 6 | Onsite | 1.5 | 3.8 | 8.9 | 11.6 | 13.1 |
| | Remote | 1.8 | 4.7 | 11.4 | 15.4 | 17.9 |
| 7 | Onsite | 0.7 | 2.1 | 7.1 | 11.8 | 14.9 |
| | Remote | 1.1 | 3.2 | 10.9 | 18.7 | 24.5 |
| 8 | Onsite | 0.7 | 2.0 | 6.7 | 11.0 | 13.6 |
| | Remote | 2.6 | 3.6 | 7.2 | 11.8 | 16.4 |

Table S4: True parameter settings for evaluating linear dose-response curves through scenarios S1.1 to S1.6.

| Scenario | $\phi_{l,0}$ ($\delta_{l,0}$) | $\phi_{l,1}$ ($\delta_{l,1}$) | $k(d)$ ($z(d)$) | $\sigma_s^2$ ($(\sigma_s^*)^2$) | $\sigma_e^2$ ($(\sigma_e^*)^2$) |
|---|---|---|---|---|---|
| S1.1 | -2.5 (0) | -2 (0) | 2 (0) | 36 (0) | 25 (0) |
| S1.2 | -2.5 (0) | -2 (0) | 2 (0) | 36 (13) | 25 (11) |
| S1.3 | -2.5 (0.5) | -1.5 (0.5) | 2 (0.5) | 36 (13) | 25 (11) |
| S1.4 | -2.5 (-0.5) | -1.5 (-0.5) | 2 (0∼2)* | 36 (13) | 25 (11) |
| S1.5 | 0 (0) | -2 (0.3) | 1 (-0.5) | 64 (36) | 36 (28) |
| S1.6 | 0 (0) | -2 (-0.3) | 1 (0.5∼1)† | 64 (36) | 36 (28) |

* In Scenario S1.4, $z(d)$ varies by dose, i.e., $z(0) = 0,\ z(1) = 0,\ z(2) = 1,\ z(3) = 1.5,\ z(4) = 2$.
† In Scenario S1.6, $z(d)$ varies by dose, i.e., $z(0) = 0.5,\ z(1) = 0.5,\ z(2) = 1.0,\ z(3) = 1.0,\ z(4) = 1.0$.

Table S5: True parameter settings for evaluating quadratic dose-response curves through scenarios S2.1 to S2.6.

| Scenario | $\phi_{q,0}$ ($\delta_{q,0}$) | $\phi_{q,1}$ ($\delta_{q,1}$) | $\phi_{q,2}$ ($\delta_{q,2}$) | $k(d)$ ($z(d)$) | $\sigma_s^2$ ($(\sigma_s^*)^2$) | $\sigma_e^2$ ($(\sigma_e^*)^2$) |
|---|---|---|---|---|---|---|
| S2.1 | 0 (0) | -1 (0) | -0.05 (0) | 2 (0) | 36 (0) | 25 (0) |
| S2.2 | 0 (0) | -1 (0) | -0.05 (0) | 2 (0) | 36 (13) | 25 (11) |
| S2.3 | 0 (0.5) | -0.8 (0.30) | -0.03 (0) | 2 (0.5) | 36 (13) | 25 (11) |
| S2.4 | 0 (-0.5) | -0.8 (-0.12) | -0.03 (-0.005) | 1 (-0.5) | 36 (13) | 25 (11) |
| S2.5 | 0 (0) | -1.5 (0.15) | -0.03 (0.020) | 1 (-0.5) | 64 (36) | 36 (28) |
| S2.6 | 0 (-0.5) | -1.5 (-0.15) | -0.03 (0) | 1 (0.5∼1)* | 64 (36) | 36 (28) |

* In Scenario S2.6, $z(d)$ varies by dose, i.e., $z(0) = 0.5,\ z(1) = 0.5,\ z(2) = 1.0,\ z(3) = 1.0,\ z(4) = 1.0$.

Table S6: True parameter settings for scenarios S3.1 to S3.12.

| Scenario | $\phi_{q,0}$ | $\phi_{q,1}$ | $\phi_{q,2}$ | Scenario | $a_1$ | $a_2$ | Scenario | Construction |
|---|---|---|---|---|---|---|---|---|
| S3.1 | -6.33 | -2.36 | 0.08 | S3.7 | 0.32 | 0.28 | S3.13 | S3.1 × S3.7 |
| S3.2 | -5.90 | -2.54 | 0.08 | S3.8 | 0.35 | 0.35 | S3.14 | S3.2 × S3.8 |
| S3.3 | -2.50 | -2.93 | 0.08 | S3.9 | 0.40 | 0.40 | S3.15 | S3.3 × S3.9 |
| S3.4 | -2.50 | -3.10 | 0.08 | S3.10 | 0.50 | 0.50 | S3.16 | S3.4 × S3.10 |
| S3.5 | -2.50 | -2.51 | 0.08 | S3.11 | 0.25 | 0.25 | S3.17 | S3.5 × S3.11 |
| S3.6 | -2.50 | -2.30 | 0.08 | S3.12 | 0.20 | 0.20 | S3.18 | S3.6 × S3.12 |

A: Visit schema 1, consisting of 12 total visits, with 7 conducted as decentralized visits.

| | V1 | V2 | V3T | V4 | V5T | V6T | V7 | V8T | V9T | V10T | V11T | V12 |
|---|---|---|---|---|---|---|---|---|---|---|---|---|
| Days (±3) | 7 | 14 | 21 | 35 | 49 | 63 | 91 | 119 | 147 | 175 | 203 | 231 |

B: Visit schema 2, consisting of 8 total visits, with 5 conducted as decentralized visits.

| | V1 | V2T | V3T | V4 | V5T | V6T | V7T | V8 |
|---|---|---|---|---|---|---|---|---|
| Days (±3) | 11 | 22 | 44 | 66 | 107 | 148 | 189 | 231 |

C: Visit schema 3, consisting of 12 total visits, with 4 conducted as decentralized visits.

| | V1 | V2 | V3T | V4 | V5 | V6T | V7 | V8 | V9T | V10 | V11T | V12 |
|---|---|---|---|---|---|---|---|---|---|---|---|---|
| Days (±3) | 7 | 14 | 21 | 35 | 49 | 63 | 91 | 119 | 147 | 175 | 203 | 231 |

D: Visit schema 4, consisting of 15 total visits, with 9 conducted as decentralized visits.

| | V1 | V2 | V3T | V4T | V5 | V6T | V7T | V8 | V9T | V10T | V11 | V12T | V13T | V14T | V15 |
|---|---|---|---|---|---|---|---|---|---|---|---|---|---|---|---|
| Days (±3) | 6 | 12 | 18 | 24 | 35 | 46 | 58 | 70 | 93 | 116 | 139 | 162 | 185 | 208 | 231 |

Figure S1: Visit schema considered in the paper: Schema 1 is used for the real-world application and main simulation study, while schema 2 to 4 are utilized for sensitivity analyses. The visits in red denote decentralized measurements.

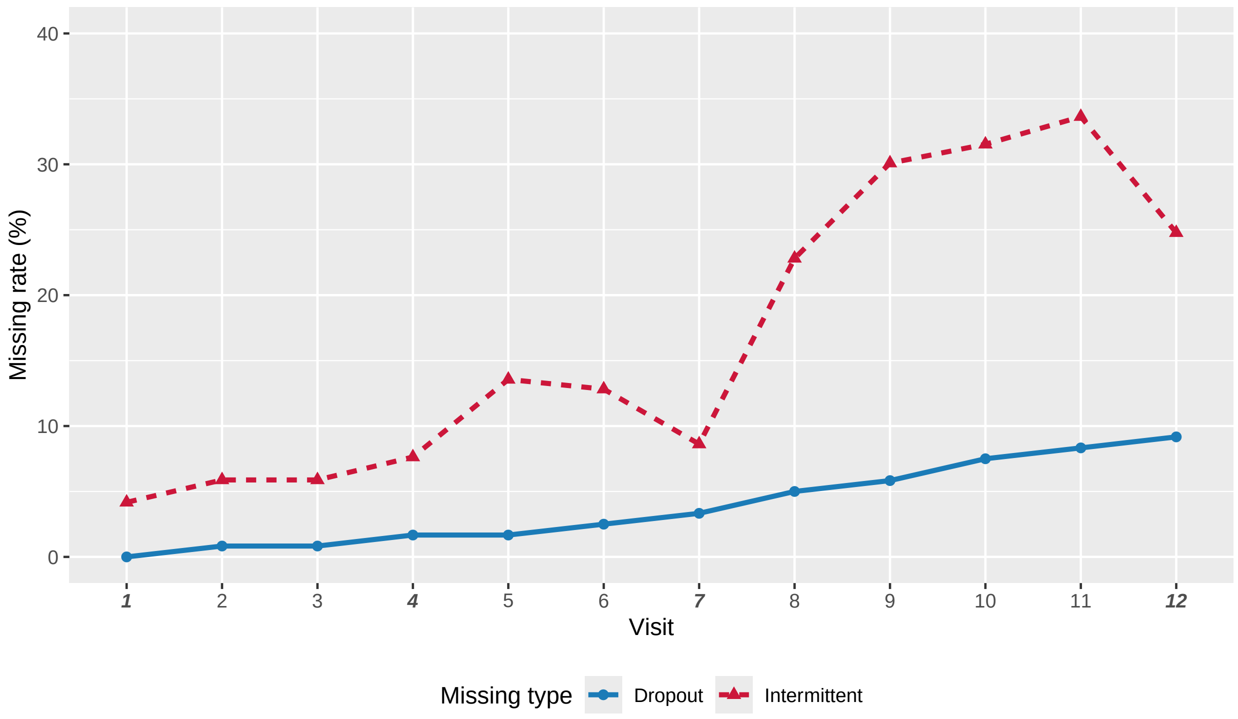


Figure S2: The cumulative dropout rates and intermittent missing rates over time for the real-world application. Centralized measurements are highlighted in bold and italics on the x-axis.

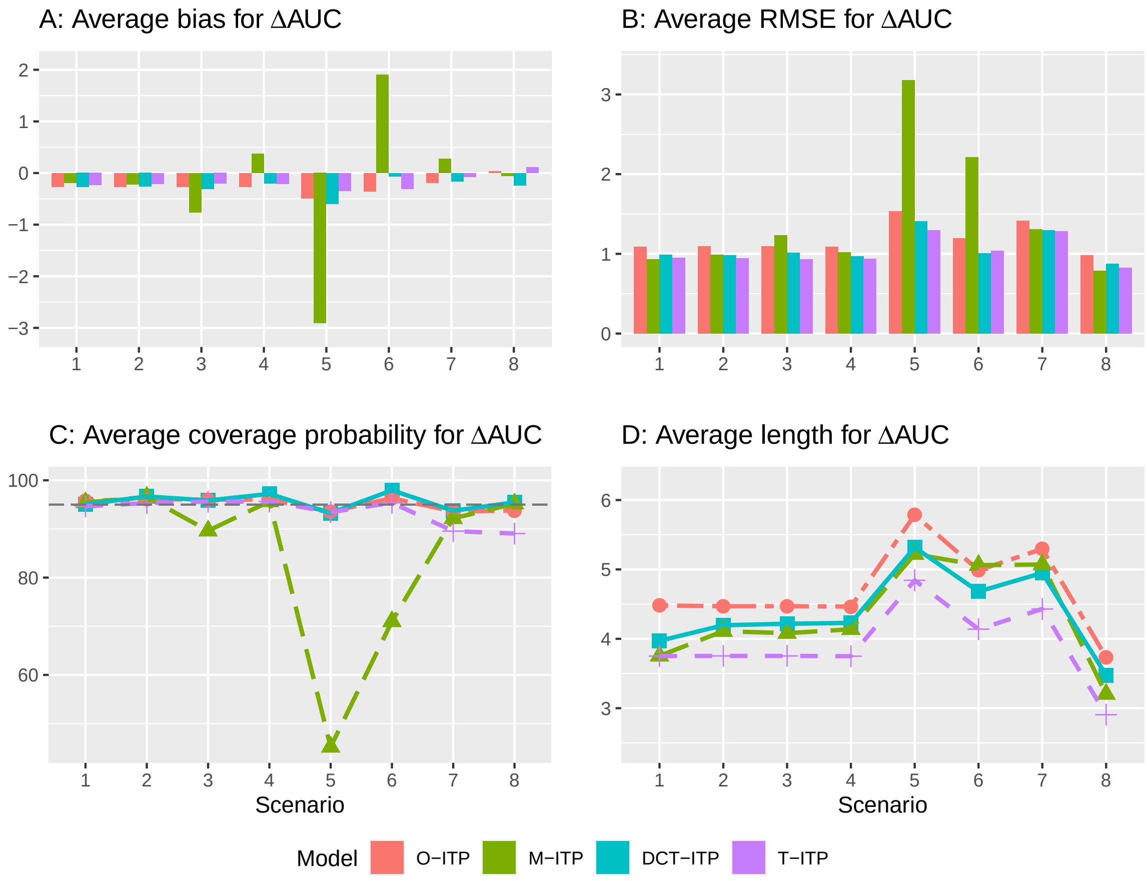


Figure S3: Average bias, average root mean square error (RMSE), and average coverage probability and length of pointwise 95% credible intervals for the estimate of the difference in the area under the time-response curve ($\Delta$AUC) using the four integrated two-component prediction (ITP) models under Scenarios 1 to 8.

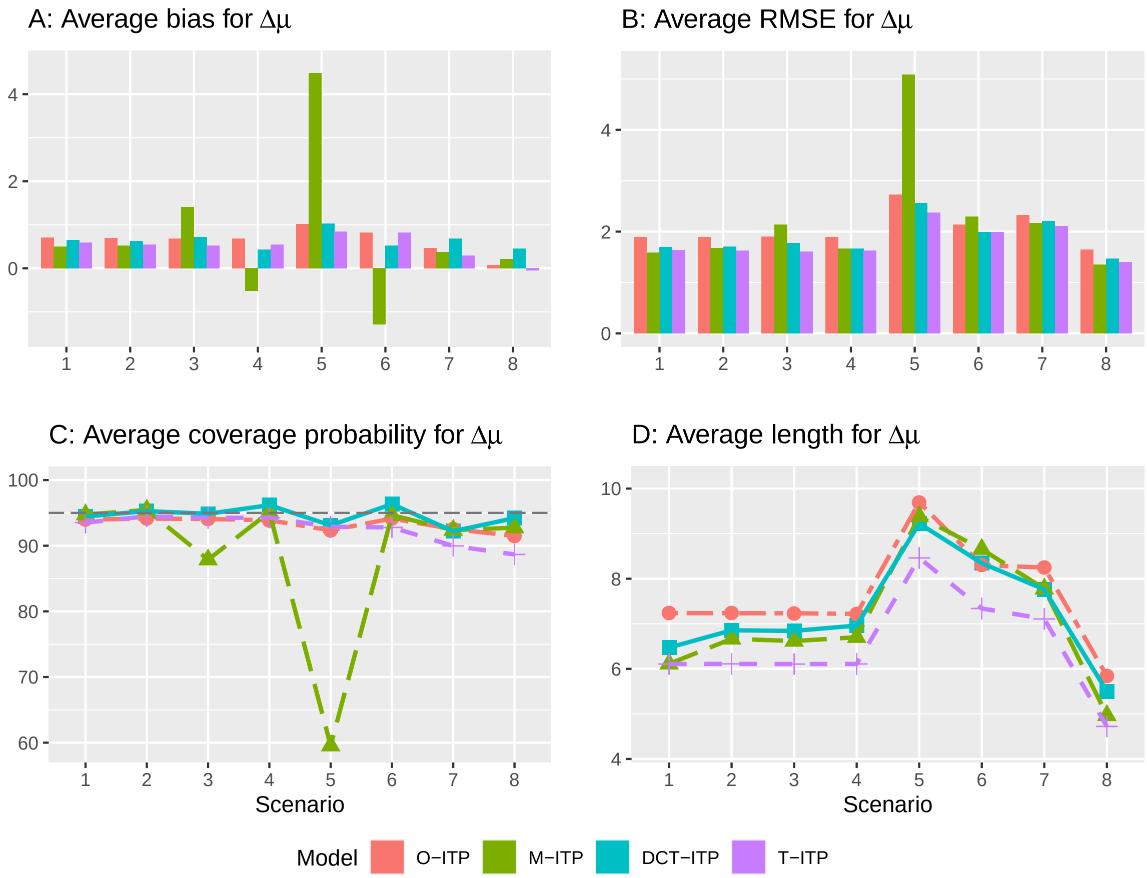


Figure S4: Average bias, average root mean square error (RMSE), and average coverage probability and length of pointwise 95% credible intervals for the estimate of the difference in the mean response at the final visit ($\Delta\mu$) using the four integrated two-component prediction (ITP) models under Scenarios 1 to 8.

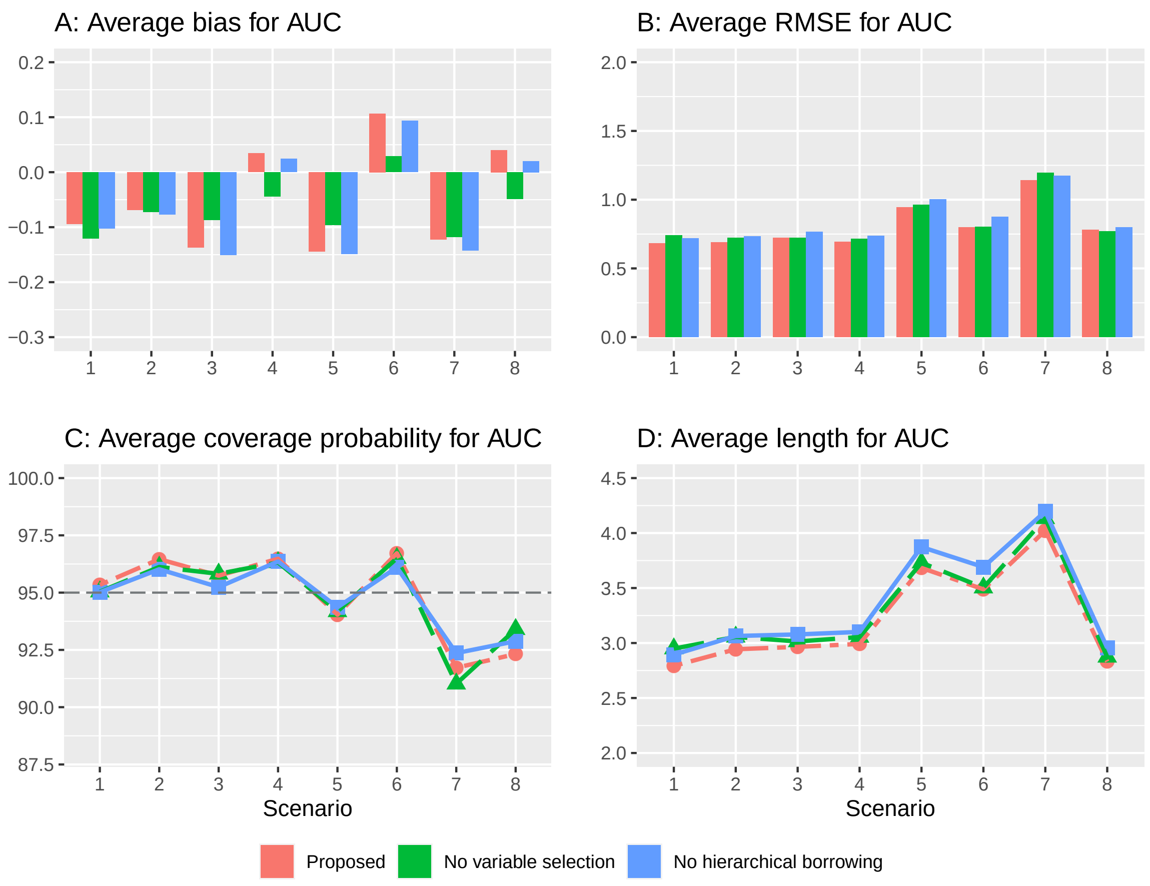


Figure S5: Average bias, average root mean square error (RMSE), and average coverage probability and length of pointwise 95% credible intervals for the estimate of the area under the time-effect curve (AUC) using the proposed model, a simplified model without Bayesian variable selection, and a simplified model without hierarchical borrowing across time-effect coefficients under Scenarios 1 to 8.

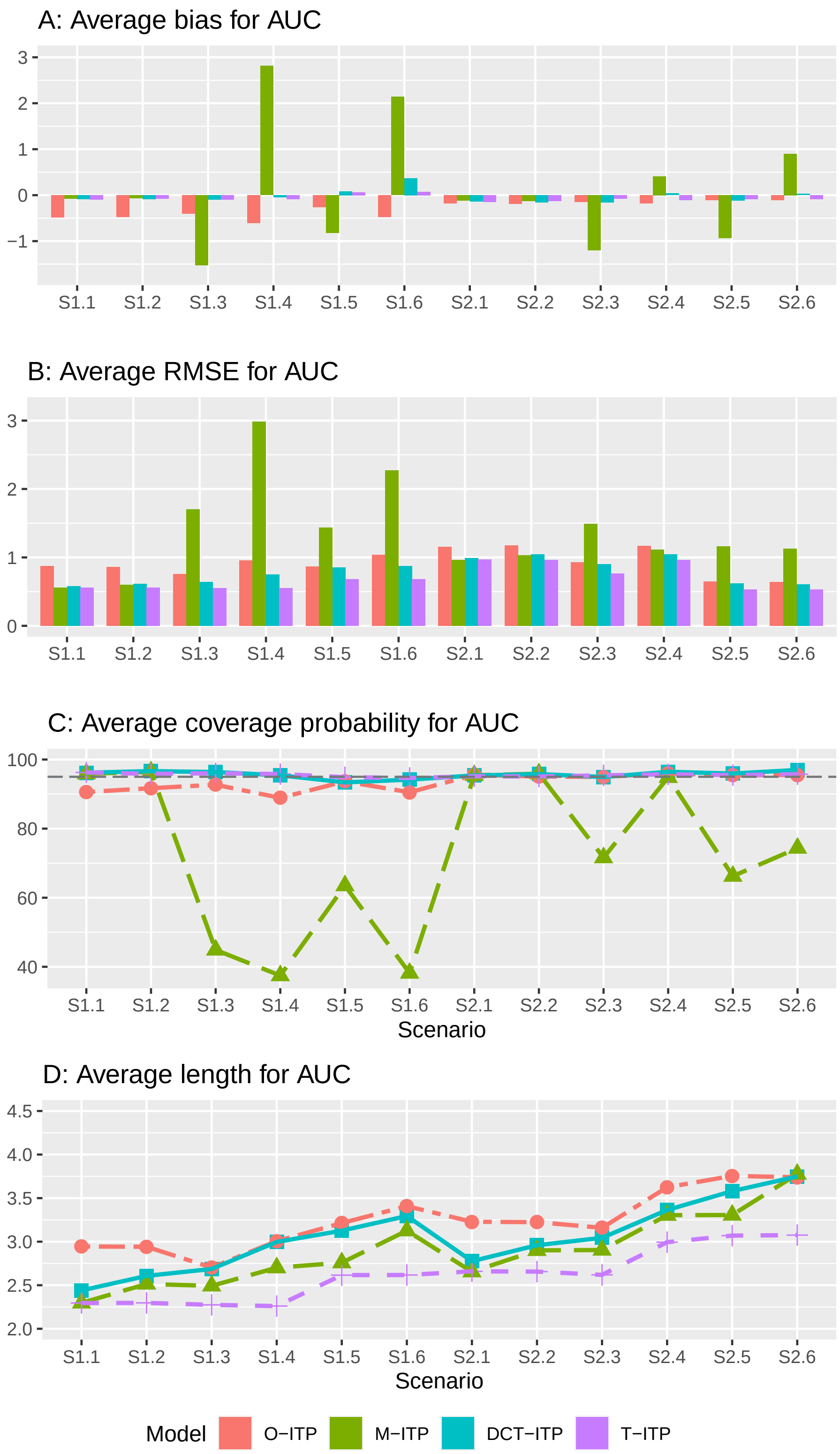


Figure S6: Average bias, average root mean square error (RMSE), and average coverage probability and length of pointwise 95% credible intervals for the estimate of the area under the time-effect curve (AUC) using the four integrated two-component prediction (ITP) models under Scenarios S1.1 to S1.6 (with linear dose-response functions) and S2.1 to S2.6 (with quadratic dose-response functions)

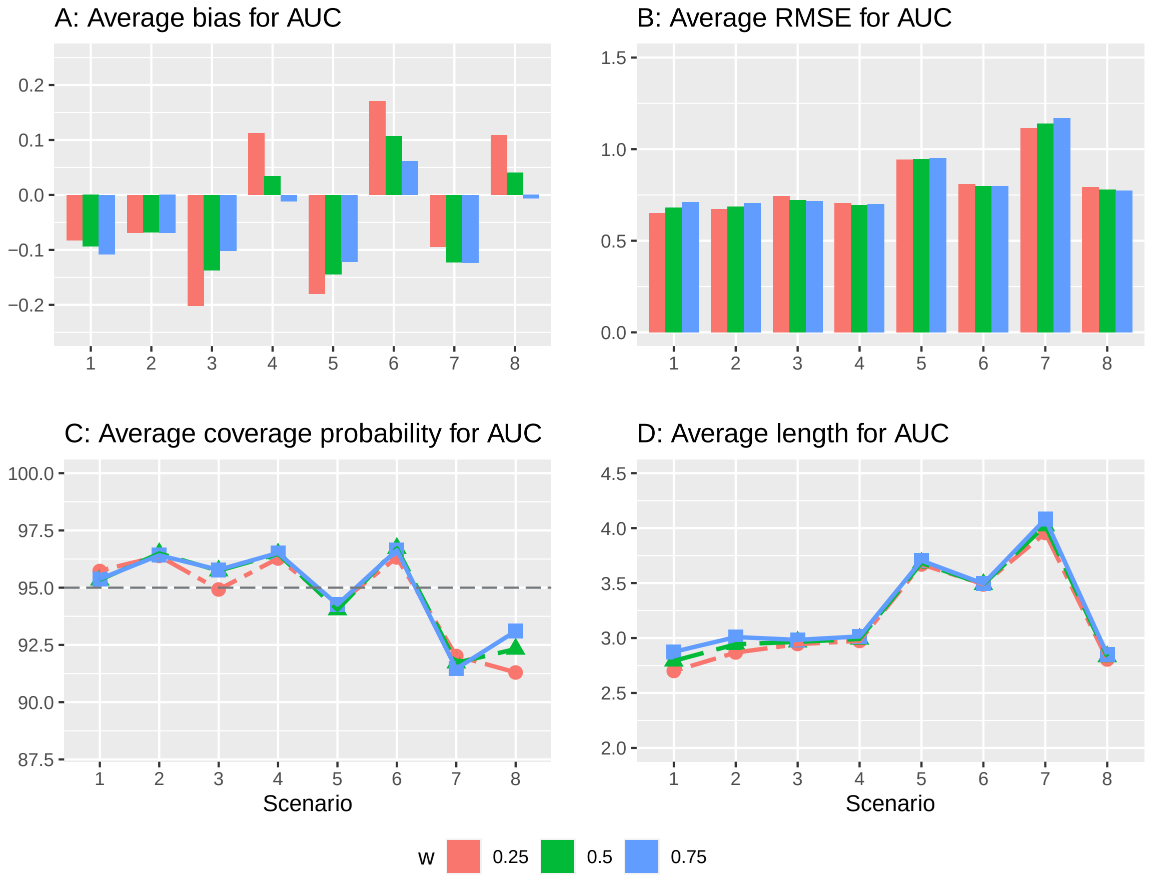


Figure S7: Simulation results for the estimated area under the time-effect curve (AUC) using the proposed DCT-ITP model, obtained under different Bernoulli prior distributions for $w_\kappa$ and $w_h$ $(h = 0, 1, 2)$, i.e., $w_\kappa, w_h \sim \text{Ber}(w)$ with $w \in \{0.25, 0.5, 0.75\}$.

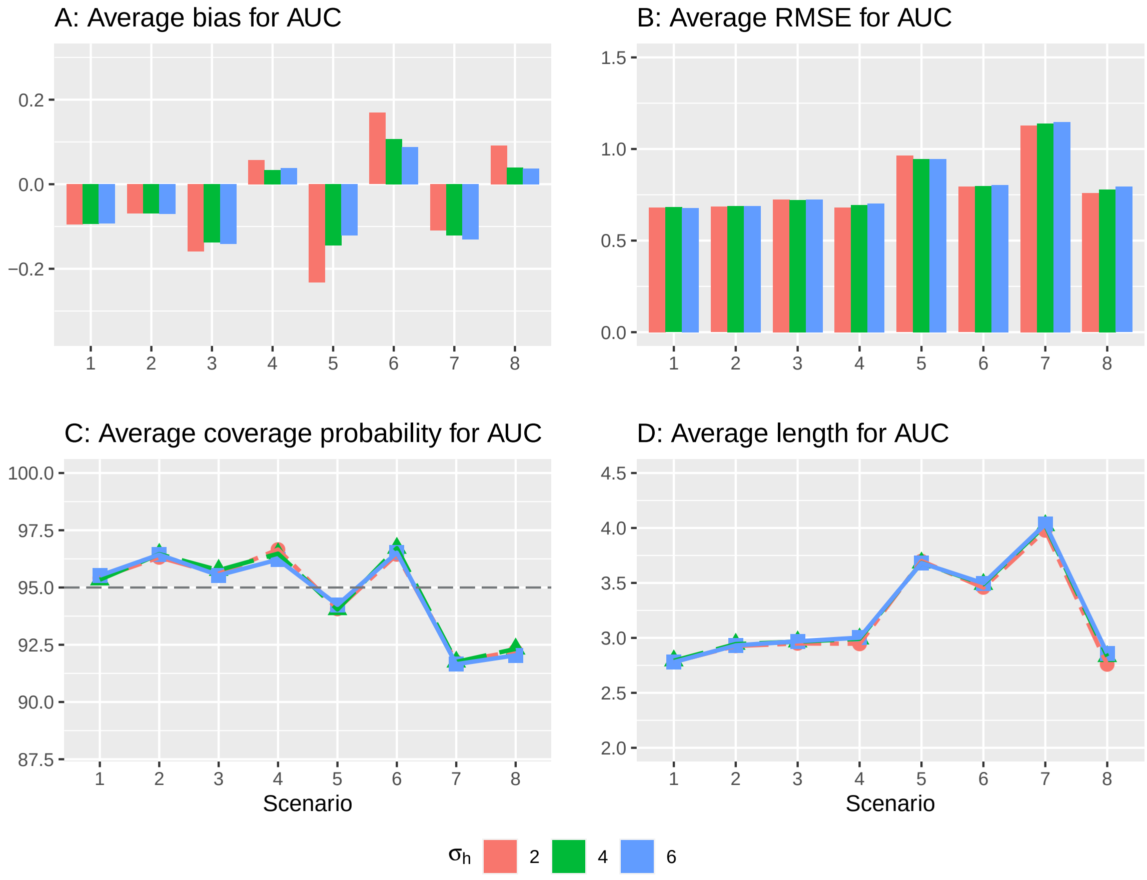


Figure S8: Simulation results for the estimated area under the time-effect curve (AUC) using the proposed DCT-ITP model, obtained under different variances of the slab component, with $\sigma_h^2 \in \{2^2, 4^2, 6^2\}$.

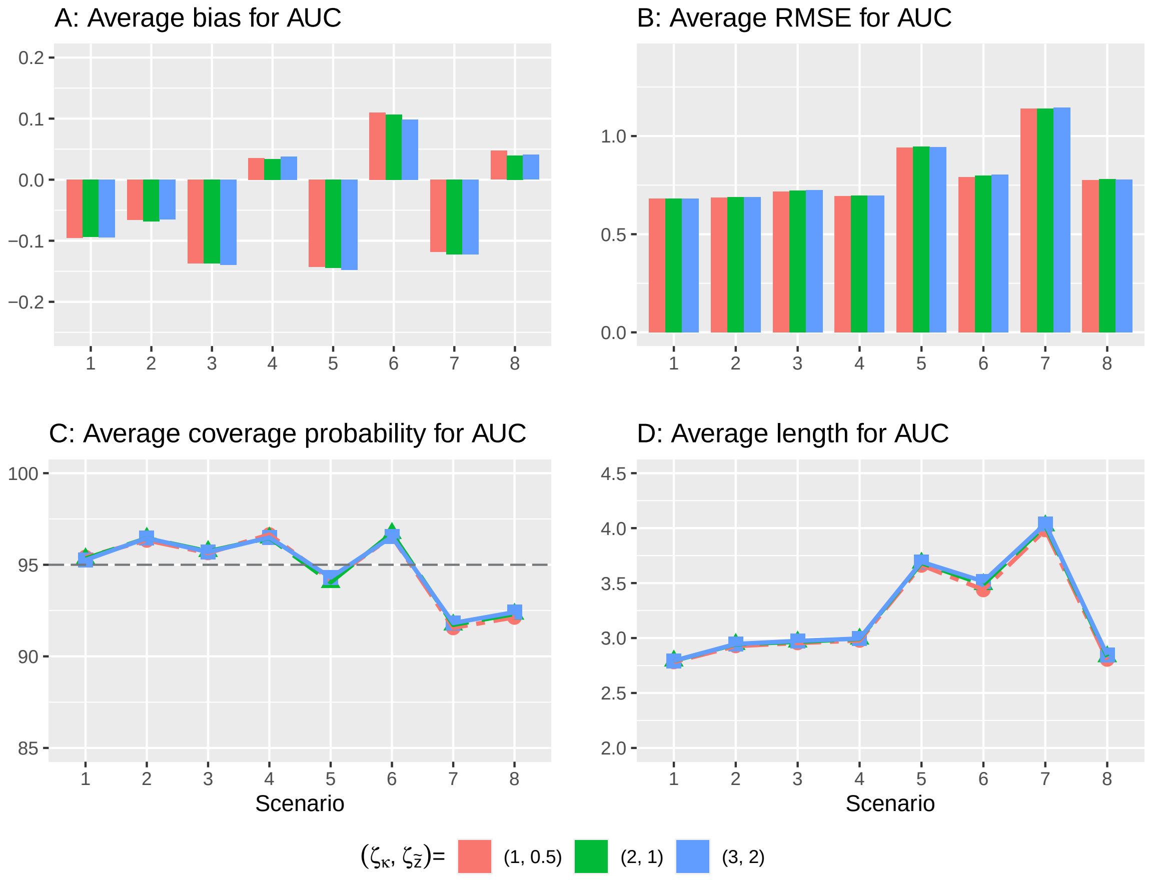


Figure S9: Simulation results for the estimated area under the time-effect curve (AUC) using the proposed DCT-ITP model, obtained under different prior scales ($\zeta_\kappa$ and $\zeta_{\tilde{z}}$) on the heterogeneity parameters, with $(\zeta_\kappa, \zeta_{\tilde{z}}) = (1,\ 0.5),\ (2,\ 1)$, or $(3, 2)$.

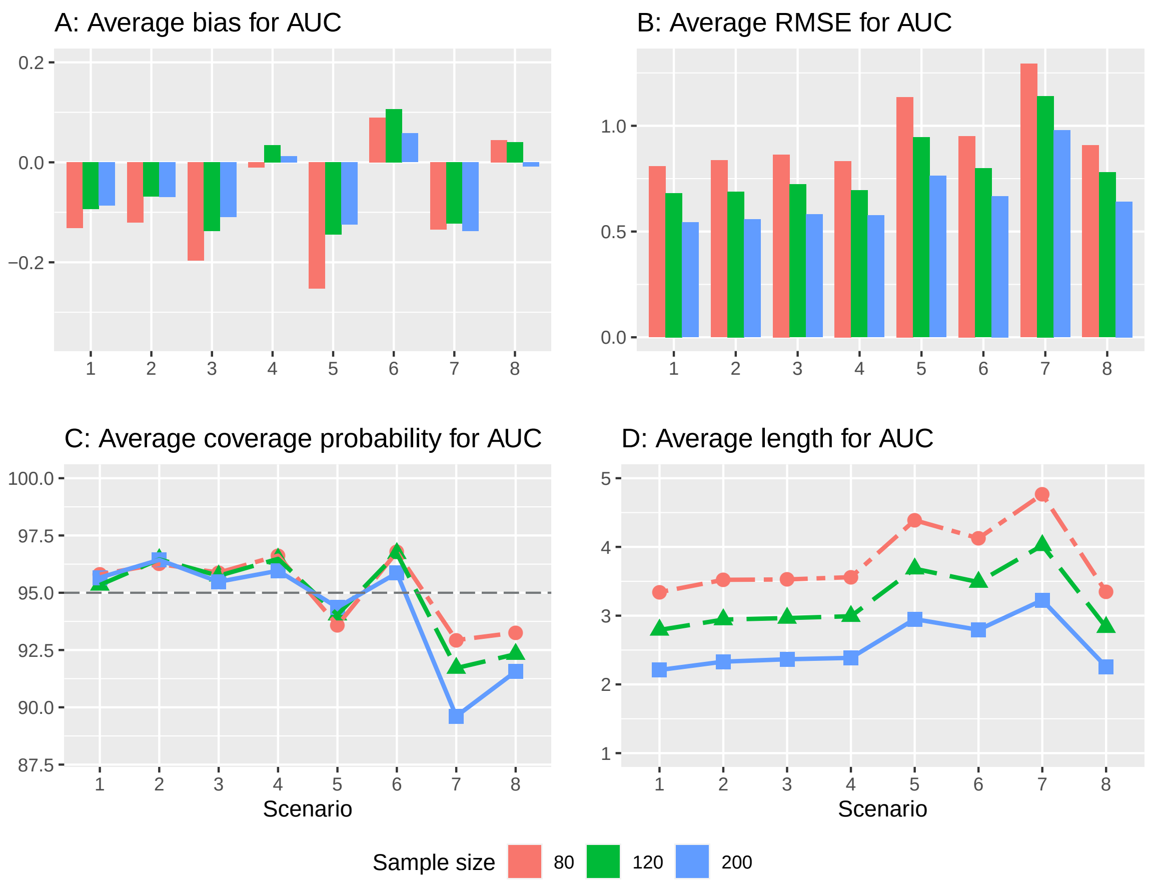


Figure S10: Simulation results for the estimated area under the time-effect curve (AUC) using the proposed DCT-ITP model based on various total sample sizes (80, 120, 200).

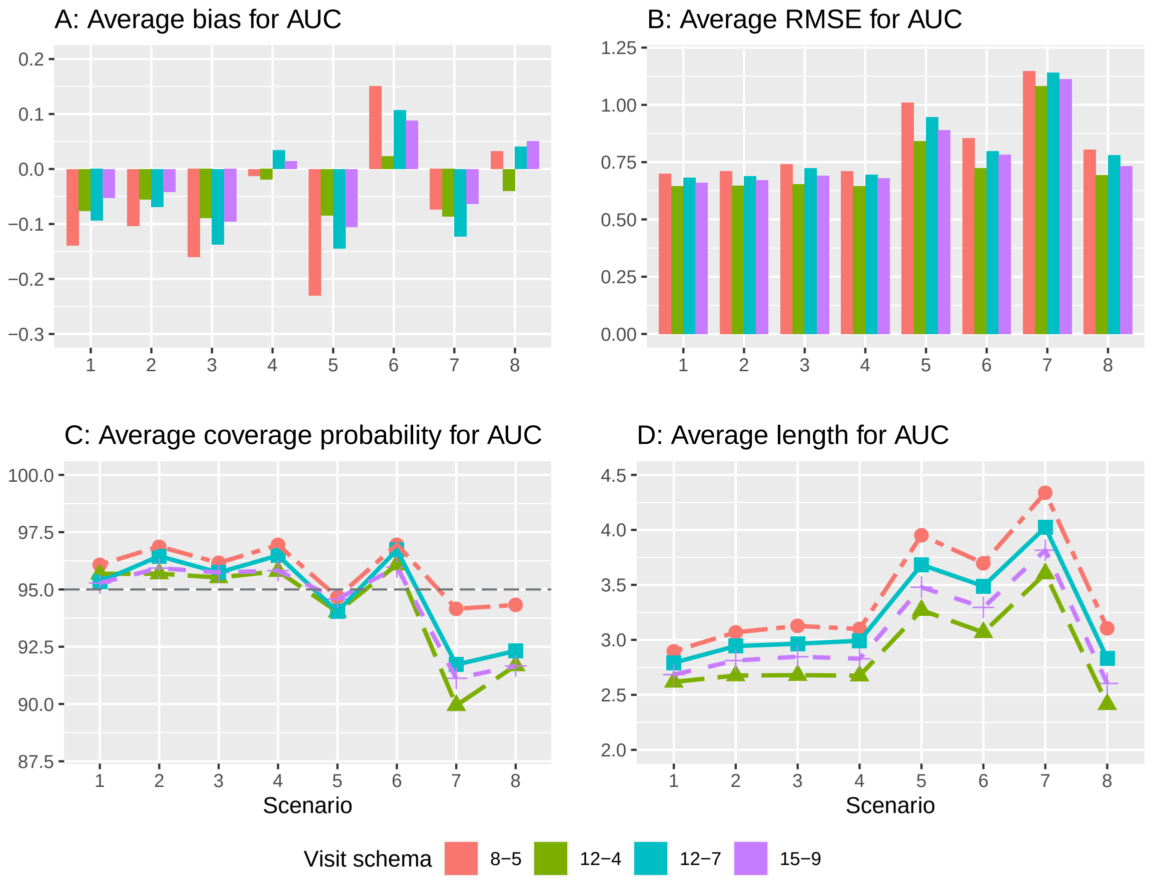


Figure S11: Simulation results for the estimated area under the time-effect curve (AUC) using the proposed DCT-ITP model based on various visit schemas. Here, "a-b" indicates that there are a total of $a$ visits and $b$ visits out of the $a$ visits are decentralized. See Figure S1 for the detailed arrangements for visit schemas.

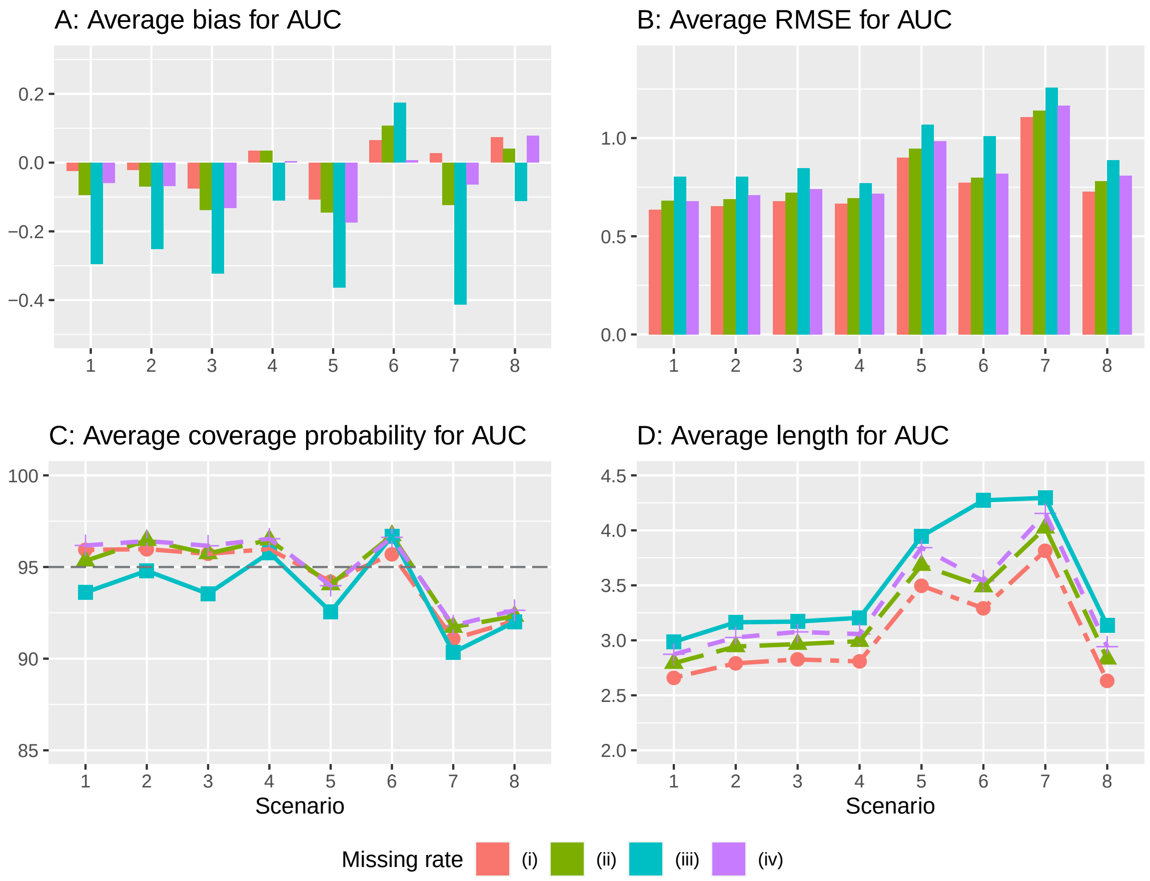


Figure S12: Simulation results for the estimated area under the time-effect curve (AUC) using the proposed DCT-ITP model based on various missing data generation mechanisms. (i)–(iii) represent the mixture of intermittent MAR and dropout, with the total average missing rate at each visit at about 10%, 20%, and 30%, respectively. (iv) represents the mixture of intermittent MCAR, intermittent MAR, and dropout at random, with the total average missing rate of 30%.

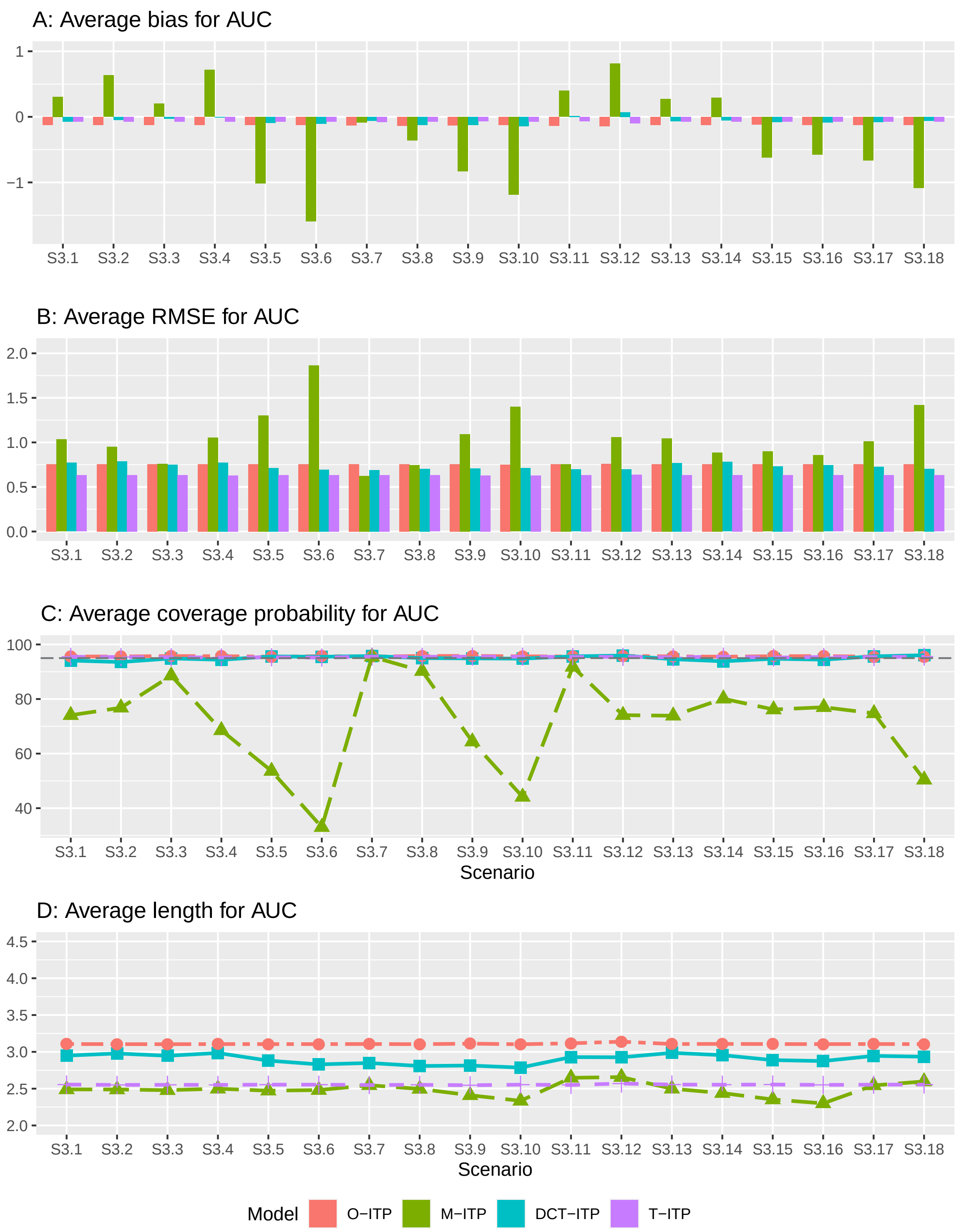


Figure S13: Simulation results for the estimated area under the time-effect curve (AUC) using the four integrated two-component prediction (ITP) models under Scenarios S3.1 to S3.18.

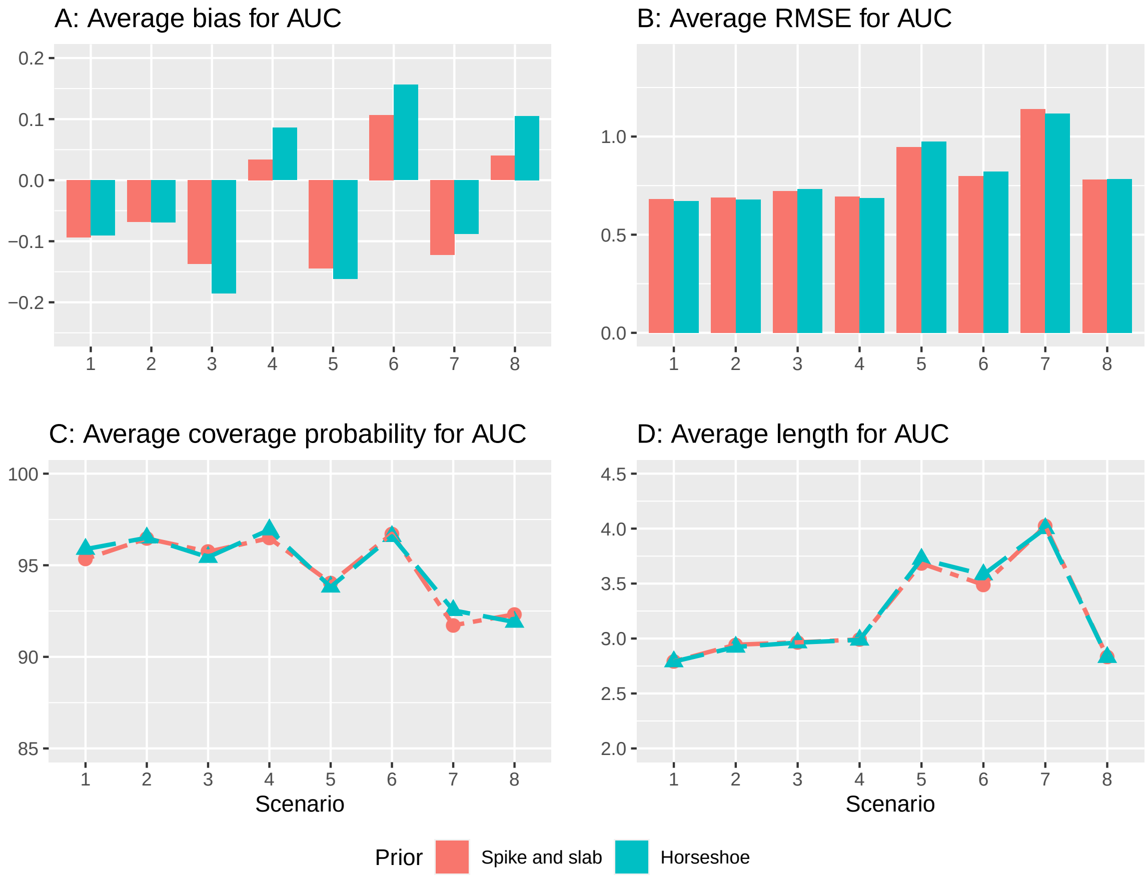


Figure S14: Simulation results for the estimated area under the time-effect curve (AUC) using the proposed DCT-ITP model using the spike-and-slab prior and the horseshoe prior.
